# Automatic Generation of Even-Tempered Auxiliary Basis Sets with Shared Exponents for Density Fitting

**DOI:** 10.1021/acs.jctc.4c01555

**Published:** 2025-02-27

**Authors:** Manuel Díaz-Tinoco, Roberto Flores-Moreno, Bernardo A. Zúñiga-Gutiérrez, Andreas M. Köster

**Affiliations:** † Departamento de Química, CINVESTAV, Avenida Instituto Politécnico Nacional 2508 A.P. 14-740, Mexico, Distrito Federal 07000, Mexico; ‡ Departamento de Química, Universidad de Guadalajara. Blvd. Marcelino García Barragán 1421, Guadalajara, Jalisco C.P. 44430, Mexico

## Abstract

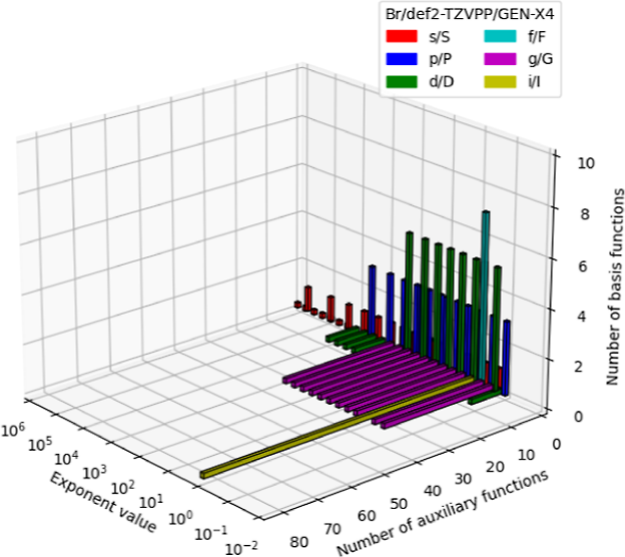

A new algorithm for the automatic generation of auxiliary basis sets for the variational density fitting (DF) of two-electron Coulomb repulsion and Fock exchange energies is presented. It generates even-tempered primitive Hermite Gaussian auxiliary basis sets with shared exponents according to the underlying orbital basis set. To this end, the auxiliary basis sets, denoted GEN-X2, GEN-X3 and GEN-X4, span the product space of the primary orbital basis set for each element. The accuracy of the GEN-X*n* (*n* = 2, 3 and 4) auxiliary basis sets was tested with the DZVP, 6-31G** and def2-TZVPP orbital basis sets for elements from H to Kr employing a large set of small molecules representing (nearly) each element in its common oxidation states. DF errors below 1 kcal/mol were reached for all systems. Whereas this fitting precision is reached in DF PBE calculations already with the smallest GEN-X2 auxiliary basis set for all test systems corresponding DF Hartree–Fock calculations require GEN-X3 and GEN-X4 for molecules containing second and third row elements (including transition metals), respectively. Most satisfying, in all cases the DF error signs reflect the theoretical variational bounds of the Coulomb and Fock fitting. The computational efficiency of the GEN-X*n* auxiliary basis sets was benchmarked by single-point energy calculations of hydrogen saturated MFI zeolite cutouts with up to 1408 atoms.

## 1 Introduction

Density fitting (DF) has a longstanding history in Kohn–Sham density functional theory (DFT)^1,2^ methods.^3,4^ A drawback of earlier approaches was their nonvariational formulation which hampered accurate energy gradient and higher energy derivative calculations.^5,6^ The variational fitting of the Coulomb potential by Dunlap and co-workers^7−9^ overcame this limitation for the two-electron Coulomb repulsion energy. As a result, variational density fitting became a standard approach in Kohn–Sham DFT codes like deMon^10^ and DGAUSS^11^ that employed the linear combination of Gaussian type orbital (LCGTO) approximation for energy and energy derivative calculations.^12^ The formal cubic scaling of these LCGTO–DFT codes drew attention to variational density fitting in the more traditional quantum chemistry community, too.^13^ Under the synonym resolution of the identity (RI), it was introduced together with Kohn–Sham DFT implementations into some quantum chemistry software packages.^14^ For the discussion of the relation between RI with variational density fitting we refer the interested reader to the literature.^15,16^ A result of the RI formulation of variational density fitting was its connection to molecular integral approximations.^17^ To this end, the importance of the positive semidefiniteness of the second order error functional proposed by Dunlap et al.^7^ was recognized as critical ingredient for the variational nature of density fitting approaches.

**Figure 1:**
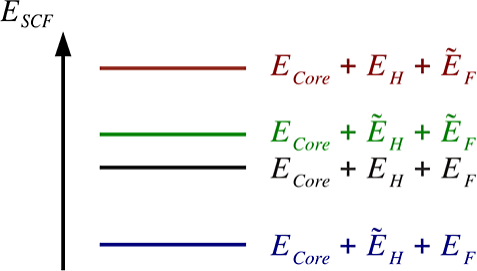
Systematic error cancellation in the variational fitting of the Coulomb and Fock energies. *E*_core_, *E*_H_, *E*_F_ denote the one-electron core, two electron Coulomb repulsion and Fock exchange energies, respectively. Fitted energies are denoted by tildes.

Whereas the variational density fitting of the Coulomb potential had already gained wide spreading acceptance two decades ago, the extension of density fitting to Fock exchange remained more controversial. This was due to the fact that its direct implementation^18,19^ led to algorithms only favorable for medium-sized molecules calculated with large orbital basis sets (OBS).^20^ To overcome this performance bottleneck Polly et al. developed the local density-fitting (LDF) for Fock exchange^21^ exploiting the short-range nature of Fock exchange. This approach was brought onto variational ground by the variational LDF for Fock exchange.^22^ In this case, the negative semidefiniteness of the underlying error functional is crucial for the variational nature of this fitting approach. A direct consequence from the variational fitting of both, Coulomb and Fock energy, is systematic error cancellation in self-consistent field (SCF) calculations as depicted in Figure 1. To this end, the same auxiliary basis set (ABS) must be used for both expansions. To ensure variational density fitting the numerical stable solution of the Coulomb fitting equation is mandatory. To this end the ill-conditioning of the Coulomb matrix due to finite precision arithmetic errors must be properly addressed. Possible algorithms are given by the truncated eigenvalue decomposition of the Coulomb matrix^23^ and Krylov methods for the solution of the fitting equation system^24^ in Section 2.1. Together with the LDF for Fock exchange, this defines variational density fitting. It is characterized by obeying variational bounds, stable SCF convergence and energy derivatives as well as excellent computational performance. For these reasons, variational density fitting has become a method of choice for three-center Electron Repulsion Integral (ERI) implementations of Hartree–Fock^21,22^ (HF) and hybrid functional Kohn–Sham DFT methods.^25,26^ While the variational density fitting has gained considerable attention over the last decades the structure and form of the underlying ABS has been little explored. Most often, it is assumed that the auxiliary basis functions are of the same type as the orbital basis functions and follow a similar structure. However, this is neither necessary nor beneficial for the variational fitting of the Coulomb and Fock energies. In fact, the structure and form of the ABS may significantly impact the computational performance of variational density fitting.

The benefit of primitive auxiliary basis functions with shared exponents for the recursive calculation of three-center ERIs has been explored many years ago.^27^ It is used in deMon2k^28^ for the variational density fitting of Coulomb repulsion and Fock exchange energies in serial and parallel calculations. As a result, auxiliary basis functions are not only grouped into shells, i.e. functions that share the same exponent and angular momentum index, but also into sets that consist of primitive functions with same exponent but different angular momentum index. Such sets contain all functions up to a highest (even) angular momentum index. Take as example a *d* auxiliary function set: It contains ten auxiliary basis functions with the same exponent, namely one *s*, three *p* (*p*_*x*_, *p*_*y*_, *p*_*z*_) and six *d* (*d*_*xx*_, *d*_*xy*_, *d*_*xz*_, *d*_*yy*_, *d*_*yz*_, *d*_*zz*_) functions. This structure permits highly efficient integral recurrence relations for three-center ERIs that can be optimized in terms of FLoating POint operations (FLOPs). To this end, deMon2k utilizes a combination of the modified Obara–Saika^29,30^ and the McMurchie–Davidson^31^ algorithms. To avoid transformation recurrence relations for the auxiliary basis functions they are directly used in form of primitive Hermite Gaussian functions.^32^ We note that these Hermite Gaussian function sets cover the same Hilbert space as their Cartesian counterparts. The primitive form of the Hermite Gaussian auxiliary basis functions further facilitates the double asymptotic expansion of the three-center ERIs which yields expressions similar to fast multipole methods for point moments but does not require explicit space division.^33^ The resulting low-order scaling ERI algorithm for the variational fitting of the Coulomb potential is excellently suited for parallelization.^34^ The parallelization of the corresponding low-order scaling ERI algorithm for the variational fitting of Fock exchange scales less well with respect to the number of cores but still shows very satisfying performance.^24^ If the auxiliary density is also used for the calculation of the exchange–correlation energy and potential, as in auxiliary density functional theory (ADFT),^35^ the just described structure of the ABS will considerably accelerate the numerical integrations, too.^36^ Thus, the structure of the ABS is indeed critical for the computational performance of ADFT as well as density fitted Kohn–Sham DFT.

Already many years ago, the particular structure of the deMon2k ABS inspired their automatic generation from the used OBS.^37^ Originally, this was limited to the variational density fitting for the two-electron Coulomb energy in Kohn–Sham DFT and ADFT calculations. Because the underlying energy expressions are variational very similar accuracies as for optimized ABS were obtained.^38^ These automatically generated ABS are named GEN-A*n* with *n* = 2, 3, 4. Increasing the *n* in GEN-A*n* increases the number of sets of the auxiliary basis functions. The angularity of the GEN-A*n* sets is restricted to *s* and *d* primitive Hermite Gaussian ABS. With the implementation of the variational fitting for Fock exchange in deMon2k the automatically generated GEN-A*n* ABS were extended to GEN-An* and GEN-An** which consists of *s*, *d*, *g* and *s*, *d*, *g*, *i* sets of primitive Hermite Gaussian auxiliary basis functions. Although, these ABS structures were never intended for the fitting of Fock exchange energies they provided quantitative agreement for the three-center ERI HF^22^ and hybrid DFT^39^ energies of molecules consisting of first- and second-row elements with respect to corresponding four-center ERI calculations. Again, the automatically generated GEN-An* and GEN-An** ABS performed as well as optimized ABS.^40^ This underlines the variational nature of the three-center HF (see Figure 1) and hybrid DFT calculations. It also shows that the automatic generation of ABS is well suited for these methodologies. This is supported by other schemes for the automatically generation of ABS in the framework of RI^41−43^ and Cholesky decomposition.^44,45^

As this discussion shows, chemically relevant energies can be obtained with excellent accuracy from variational density fitted three-center ERI calculations even when their total energies differ appreciably from their four-center ERI counterparts. Nevertheless, the comparison of total energies has become a common standard.^43,46^ To adapt to this standard, we present here a new automatic generator for ABS that preserves the above-described structure of the GEN-A*n*, GEN-An* and GEN-An** sets but yields absolute energy deviations from four-center ERI calculations below 1 kcal/mol throughout the periodic table.

The manuscript is organized as follows. In the first part of Section 2 the variational density fitting formulas for the two-electron Coulomb and Fock energies are briefly reviewed. In particular, the different forms of the working equations are presented and the problem of finite precision arithmetic errors is addressed. The new automatic generator for GEN-X*n* ABS, with *n* being 2, 3 and 4, is outlined in Section 2.2. The computational methodologies for the four- and three-center ERI HF and Kohn–Sham calculations are described in Section 3. The results are discussed in Section 4 and the final conclusions are presented in the last section.

## 2 Theory

### 2.1 Variational Density Fitting

The (closed-shell) variational density fitting of the two-electron Coulomb repulsion and Fock exchange energy is based on the following two second-order error functionals
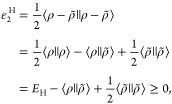
1
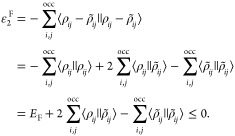
2

In these equations *E*_H_ and *E*_F_ refer to the two-electron Coulomb repulsion and Fock exchange energies, respectively. The symbol ∥ represents the two-electron Coulomb operator  in the short-hand notation of the ERIs. It also separates the functions depending from electron 1 in the left bra from those of electron 2 in the right ket. We will use analogous notations for other three-center and two-center two-electron integrals, too. The (closed-shell) density, , and orbital product densities, , are given by
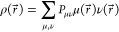
3
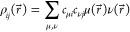
4

In eqs 3 and 4*P*_μν_ denotes the (closed-shell) density matrix
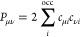
5and *c*_μ*i*_ the corresponding molecular orbital (MO) coefficients. Greek letters are used for (contracted) atomic orbitals. The fitted density, , and orbital product densities,  are expanded as
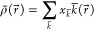
6
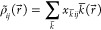
7

In eqs 6 and 7 and  are Coulomb and Fock fitting coefficients, respectively. The  are primitive Hermite Gaussian auxiliary basis functions (denoted by a bar) that are used for both expansions.

The positive semidefiniteness of ε_2_^H^, eq 1, permits the formulation of the variationally approximated two-electron Coulomb repulsion energy as
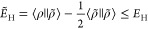
8

Expanding  and  according to eqs 3 and 6 yields the following equivalent working equations for the variational density fitted two-electron Coulomb repulsion energy
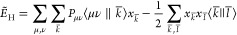
9a
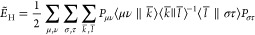
9b
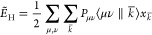
9c
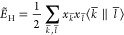
9d

In deMon2k eq 9a is used for the variational fitting of the Coulomb energy. This allows the use of a MinMax SCF approach.^23^ In any case, all four fitted Coulomb repulsion energy expressions are equivalent at SCF convergence.

Similarly, the negative semidefiniteness of ε_2_^F^, eq 2, permits the formulation of the variationally approximated Fock exchange energy as
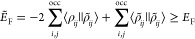
10

Expanding  and  according to eqs 4 and 7 yields the following equivalent working equations for the variational orbital density fitted Fock exchange energy
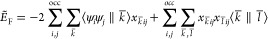
11a
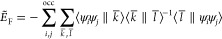
11b
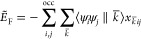
11c
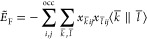
11d

In these equations, ψ_*i*_ and ψ_*j*_ denote MOs. In deMon2k eq 11b is used for the variational fitting of the Fock exchange energy because it allows the use of localized MOs. The resulting LDF exploits the short-range nature of Fock exchange. As a result, the inverse Coulomb matrix elements, , in eq 11b are only built from a local subset of the used auxiliary basis functions. In practice, they are obtained from a truncated eigenvalue decomposition (TED) or modified Cholesky decomposition of the local Coulomb matrices in deMon2k.^24^ Because of the small size of these matrices this decomposition is of no concern for the computational performance, even for very large systems. Again, at SCF convergence, all four fitted Fock exchange energy expressions are equivalent.

Unfortunately, the situation is different for the fitted Coulomb energy calculated with eq 9a. For the Coulomb fitting coefficients, , the following inhomogeneous equation system, with the dimension of the full ABS, must be solved
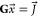
12

In eq 12**G** denotes the Coulomb matrix with elements ,  collects the Coulomb fitting coefficients and  is the Coulomb vector that contains three-center ERIs. Although the Coulomb matrix is formally positive definite in practical calculations **G** can become slightly indefinite and ill-conditioned due to finite precision arithmetic errors. This can destroy the variational nature of the fit as indicated by negative MinMax errors in the SCF. Automatically generated ABS are particularly prone to this problem. Furthermore, because **G** changes with the molecular structure, a well-conditioned **G** matrix at an optimized or experimental molecular structure may turn into an ill-conditioned one during molecular dynamics simulations. Thus, a general solution for the possible ill-conditioning of **G** is needed. To this end, deMon2k offers three approaches, triggered by the keyword MATINV, for the solution of eq 12. The first one (MATINV ANALYTIC) is a TED in the diagonal basis of the Coulomb matrix which guarantees positive MinMax errors. For small systems with up to 10 000 auxiliary basis functions this is the method of choice in terms of accuracy as well as computational performance. A drawback of this approach is the diagonalization of **G** in each geometry step. To overcome this computational bottleneck for systems with more than 10 000 auxiliary basis functions a quasi-Newton approach^47^ was adapted for the variational fitting of the Coulomb energy.^48^ To ensure the positive definiteness of the Coulomb matrix a TED is performed for the initial molecular structure. The approximated inverse Coulomb matrices for the following molecular structures, e.g. in geometry optimizations or molecular dynamics simulations, are obtained by inverse Broyden–Fletcher–Goldfarb–Shanno (BFGS) updates.^49^ This approach is the default in deMon2k for solving eq 12 and performs well for systems with up to 50 000 auxiliary basis functions. From this size on the initial TED of the Coulomb matrix, which requires the diagonalization of **G**, becomes a computational bottleneck. Therefore, for large systems the Krylov method MINRES^50,51^ (triggered by MATINV MINRES) was adopted for the solution of eq 12.^52^ These algorithms for the stable solution of the fitting equation systems greatly facilitate the use of automatically generated ABS for energy and energy derivative calculations.

### 2.2 GEN-X*n* Algorithm

The GEN-X*n* algorithm described here automatically generates an ABS for a given OBS. The main objective of the GEN-X*n* algorithm is to create an even tempered ABS consisting of primitive Hermite Gaussian auxiliary basis functions that are grouped together in sets with common exponents. As already discussed, this ABS structure significantly reduces the computational effort. Here we demonstrate, in form of the GEN-X*n*, that these sets can reach fitting precision below 1 kcal/mol in the total energy with respect to corresponding four-center ERI calculations. To do so, we cover the interval of the number line spanned by the exponent products for each angularity *l* of the OBS by auxiliary function exponents with angularity *L* = 2*l* according to min(α^*L*^) ≤ 2 min(ζ^*l*^) and max(α^*L*^) ≥ 2 max(ζ^*l*^), where ζ^*l*^ is the exponent of a orbital basis function with angular momentum index *l* and α^*L*^ is the exponent of an auxiliary function with angular momentum index *L*. To ensure homogeneous covering of high angular orbital basis function products by the GEN-X*n* auxiliary basis functions we use as starting point for the even-tempered auxiliary function exponent generation . Most often  belongs to polarization functions of the OBS. For this reason, the GEN-X*n* algorithm is particularly well-suited for OBS that contain polarization functions, such as the Ahlrichs def2-TZVPP,^53^ the deMon2k DZVP,^37^ or the Pople 6-31G** OBS.^54−62^

**Figure 2:**
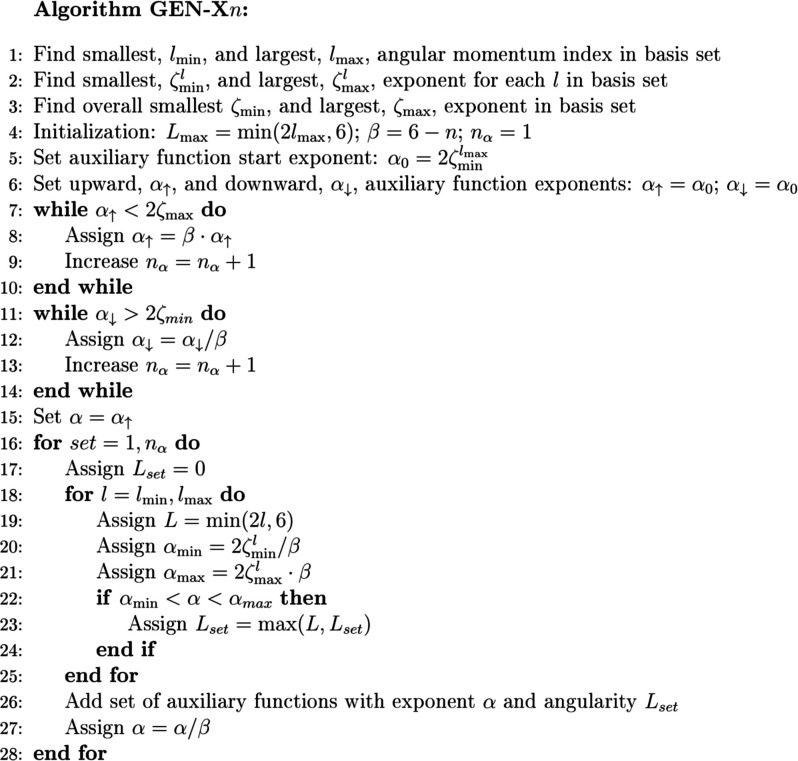
GEN-X*n* algorithm for the automatic generation of atomic auxiliary basis sets. For clarity of presentation the field index of α is omitted. The algorithm is called for each atom individually with *n* as an argument.

Figure 2 depicts step-by-step the GEN-X*n* algorithm as implemented in deMon2k. The algorithm is called for each atom individually. The first three lines determine the angularity and exponent ranges of the underlying atomic OBS. The automatic generation of the auxiliary basis functions is initialized in line 4. The largest angular momentum index for an ABS, *L*_max_, is set. In the setup used here the default upper limit of 6 is employed. This limit is given by a parameter (MAXLAUX) in deMon2k that controls the corresponding field limits in the integral calculation, too. We note that the default value of 6, i.e. auxiliary function sets until *i* angular momentum indices, is fully sufficient to achieve the aimed fitting precision with the here utilized atomic OBS. In the initialization, the scaling factor β for the generation of the even-tempered ABS is also calculated. Take as example GEN-X2 with *n* = 2. This yields β = 6 – 2 = 4. Thus, increasing *n* in GEN-X*n* reduces the spacing between auxiliary function set exponents and, therefore, leads to an increase in auxiliary basis functions. Finally, *n*_α_ initializes the counter for the generated ABS exponents and, thus, for the sets of auxiliary basis functions. As already mentioned, the start auxiliary function exponent, , is generated within the exponent range of the atomic OBS (line 5 in Figure 2) which makes an upward (line 7 to 10 in Figure 2) and downward (line 11 to 14 in Figure 2) progression of auxiliary basis functions necessary. Note that the resulting smallest auxiliary function exponent can be smaller than 2ζ_min_ and the largest auxiliary function exponent can be larger than 2ζ_max_. Through these fuzzy bounds a fully even-tempered set of auxiliary function exponents is generated.

Once the even-tempered auxiliary function exponents are determined, angular momentum indices must be assigned to them. This is done in the second part of the GEN-X*n* algorithm, from line 15 to 28 in Figure 2. To this end, the α variable is initialized with the largest auxiliary function exponent α_↑_ in line 15. The following loop, from line 16 to 28 in Figure 2 assigns to each auxiliary function exponent an angular momentum index that defines the corresponding auxiliary function set. To do so, we initialize the auxiliary function set angularity to zero, *L*_set_ = 0 in line 17 of Figure 2, i.e. to a *s*-type auxiliary function set. The next loop, line 18 to 25 in Figure 2, tests if the current processed auxiliary function exponent lies in a range of OBS product exponents, α_min_ and α_max_, with higher angular momentum index. If this is true, the auxiliary function set angular momentum index is increased to min(2*l*, 6) where 2*l* is the angular momentum index associated with the OBS product. After the angular momentum index loop, line 26 in Figure 2, we add the set with exponent α and angularity *L*_set_ to the ABS and decrease the α value by dividing it by β. In this way we keep the sets ordered from highest to lowest exponent value.

**Figure 3:**
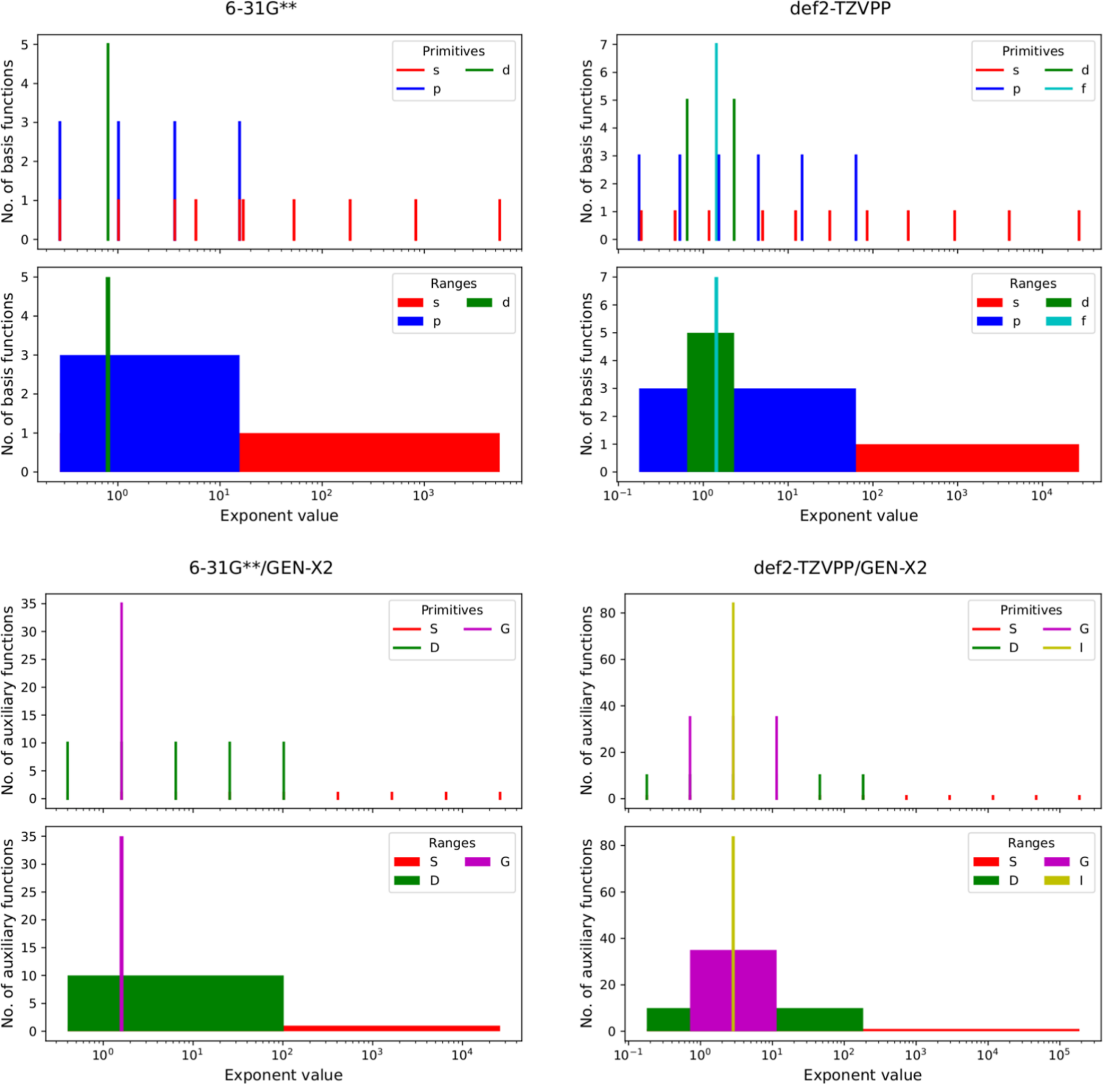
Number of basis and auxiliary basis functions vs exponent values for the 6-31G** (left) and def2-TZVPP (right) orbital basis sets of oxygen. The automatically generated GEN-X2 auxiliary basis sets are shown in the lower panels. Note the correlation between the number of auxiliary basis functions and the set angularity given by the color legends. The angularity ranges are displayed, too.

To gain insight into the structure of the automatically generated GEN-X*n* ABS we compare in Figure 3 the exponents of the 6-31G** (left) and def2-TZVPP (right) OBS of oxygen (top panels) with those of the GEN-X2 generated ABS (bottom panels). The top panels of Figure 3 show the distribution of the primitive OBS exponents and exponent ranges. The bottom panels show the corresponding distributions of the ABS exponents and exponent ranges. The heights of the bars give the number of OBS and ABS functions. As Figure 3 shows a maximum number of functions in the OBS is found in an intermediate exponent range around the primitive polarization functions. This behavior holds for all elements from H to Kr when using these two OBS. Additional plots are given in the Supporting Information. For this reason, we chose the self-product of the polarization function, i.e. the highest basis function bar, as the starting primitive, α_0_, for the generation of ABS with the GEN-X*n* algorithm. As a result, downhill and uphill progressions are needed. The resulting ABS, in terms of their primitive exponents and exponent ranges, are depicted in the lower panels of Figure 3. As for the OBS a maximum number of functions, now expressed through the high angularity of the ABS, is found in an intermediate exponent range. However, different to the OBS exponents, the primitive ABS exponents are even-tempered over the full exponent range by construction.

**Figure 4:**
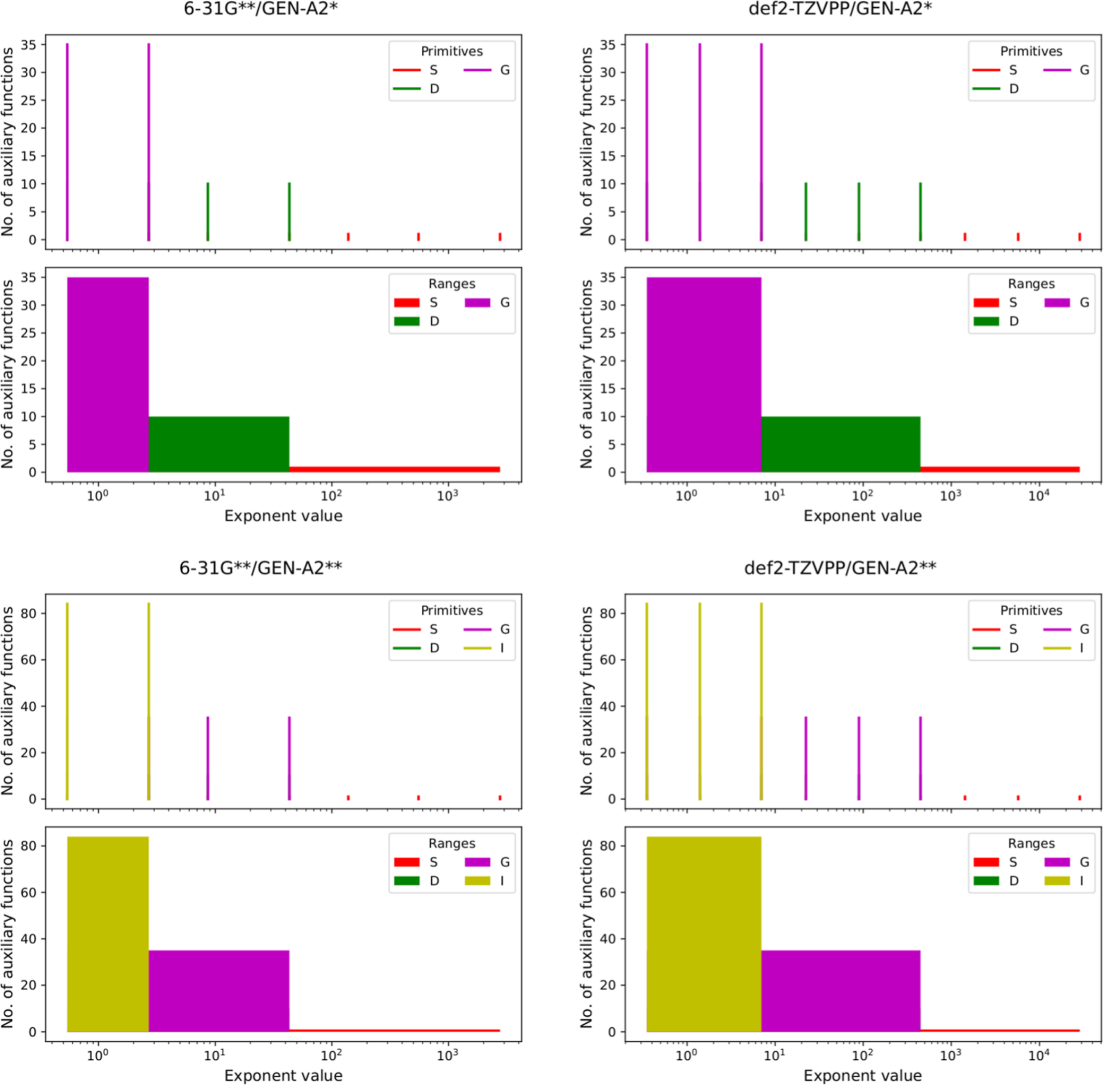
Number of auxiliary basis functions vs exponent values for the 6-31G** (left) and def2-TZVPP (right) orbital basis sets automatically generated GEN-A2* (top panels) and GEN-A2** (lower panels) auxiliary basis sets of oxygen. Note the correlation between the number of auxiliary basis functions and the set angularity given by the color legends. The angularity ranges are displayed, too.

To put the automatic generation of ABS with the GEN-X*n* algorithm in perspective, we compare it with results from GEN-An* and GEN-An** ABS generation. To this end, we depict in Figure 4 the GEN-A2* (top panels) and GEN-A2** (bottom panels) exponents and exponent ranges analog to Figure 3 for the 6-31G** (left panels) and def2-TZVPP (right panels) oxygen OBS. Although GEN-An* and GEN-An** ABS share by construction many similarities with the corresponding GEN-X*n* ABS (*L*_max_ = 6, β = 6 – *n*, shared sets of *s*, *spd*, *spdfg* and *spdfghi* primitive Hermite GTOs) comparison of Figures 4 with 3 shows marked differences. Whereas in GEN-A*n* type ABS the set angularity and, thus, the number of functions per exponent decreases monotonically from small to large exponents (see Figure 4) in GEN-X*n* type ABS the angularity starts small for small exponents, then increases to a maximum value in the range of polarization function exponents of the underlying OBS and from there on decreases monotonically again. In this way the GEN-X*n* ABS reflect the OBS angularity distribution and, thus, the number of basis function distribution, of the underlying OBS. See the Supporting Information for comparative plots for all studied elements.

## 3 Computational Methods

The new automatically generated GEN-X*n* ABS have been validated with three test sets employing the def2-TZVPP, 6-31G** and DZVP OBS. The four-center ERI reference calculations were performed using the Psi4 code.^63^ For the deMon2k three-center ERI density fitting calculations the current developer version 6.2.7^64^ was used. Three electronic structure methods were selected: HF, PBE^65,66^ and PBE0.^67^ The SCF energy convergence was set to 10^–8^ a.u. and a fine fixed grid without pruning in combination with spherical orbitals were used. For systems with problematic SCF convergence the dynamical level-shift procedure and the second-order SCF convergence method were used in deMon2k and Psi4, respectively. For these cases the number of maximum SCF cycles was increased from 100 to 500. Table 1 lists the keyword settings used to make three-center ERI deMon2k results directly comparable with four-center ERI Psi4 ones (using the scf_type pk keyword to trigger four-center ERI calculation).

**Table 1:** Keywords for deMon2k and Psi4 Input Files

deMon2k keywords	Psi4 keywords
SCFTYPE TOL = 1.0e-8	e_convergence 1.0e-8
SHIFT –0.2 MIN = 0.05^a^	soscf true^a^
GRID FIXED FINE	dft_spherical_points 590^b^
QUADRATURE UNPRUNED^b^	dft_radial_points 99

a Only for cases with problematic SCF convergence.

b Unpruned grids are used.

Total energy comparisons were performed with the test set from Weigend (T186).^38^ This test set includes 186 molecules representing nearly each element from H to Kr with their common oxidation states. Standard heats of formation (Δ*H*_f_^298K^) were obtained by the method from Curtiss et al. for the 223 molecules of the G3/99 test set.^68,69^ Molecular geometries, zero-point energies and enthalpy corrections were taken from literature B3LYP/6-31G(2df,p) calculations.^68,70^ The atomization energies (*D*_e_)
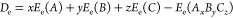
13are obtained with both, three and four-center integral codes. In eq 13*E*_e_(*A*_*x*_*B*_*y*_*C*_*z*_) denotes the energy of the molecule and *E*_e_(*A*), *E*_e_(*B*), *E*_e_(*C*) are the energies of the atoms *A*, *B*, *C*, respectively. The *x*, *y* and *z* are the stoichiometric coefficients of the corresponding elements. The G3 test set contains 223 standard heats of formation including hydrocarbons, substituted hydrocarbons, non-hydrogen molecules and radicals.^71^

To benchmark the computational efficiency of the GEN-X*n* ABS single-point energy calculations of hydrogen saturated MFI (Mobil-type FIve) zeolite cutouts were performed at the PBE/DZVP level of theory. These systems contain 376, 720, 1064, and 1408 atoms with OBS sizes ranging from around 5000 to 20 000 functions. For the variational density fitting the MINRES approach (MATINV MINRES) was used in all benchmark calculations. All benchmark calculations were performed in parallel on 96 Intel (R) Xeon (R) E5–2650 v4 2.20 GHz cores.

## 4 Results

### 4.1 Variational Density Fitting Errors in Total Energies

Table 2 lists the mean signed error (MSE) and mean absolute error (MAE) of the total SCF energy from variational fitted three-center ERI (3c-ERI) PBE, PBE0 and HF calculations with respect to corresponding four-center ERI (4c-ERI) results for the T186 test set. We present for all three OBS, def2-TZVPP, 6-31G** and DZVP, MSEs and MAEs with GEN-X*n*, GEN-An* and GEN-An** ABS. The γ̅ in Table 2 denotes the average ratio between the number of auxiliary and basis functions for a given OBS and ABS combination. As larger γ̅ as larger is the prefactor in the formal cubic scaling of 3c-ERI calculations. Table 2 shows that all MSE for PBE are negative, whereas all MSE for HF are positive as expected from Figure 1 for variational density fitting. Furthermore, the absolute MSE values for PBE and HF match within 0.01 kcal/mol the corresponding MAEs which underlines the stability of the variational density fitting in deMon2k. Note that for PBE0 that mixes Fock and PBE exchange slightly larger deviations between absolute MSE and MAE values are found. Table 2 also shows that the GEN-An* ABS are very efficient (3.6 ≤ γ̅ ≤ 6.7 for def2-TZVPP) but attain fitting precision ≲1 kcal/mol only for the PBE calculations. When using HF or PBE0, the GEN-An** or GEN-X*n* ABS can yield fitting precision below 1 kcal/mol with *n* > 2. However, the GEN-X*n* is more efficient than the GEN-An** ABS and yields excellent results when paired with the def2-TZVPP OBS. For the smaller 6-31G** and DZVP OBS the GEN-X*n* accuracies deteriorate slightly. The reason for this fitting precision deterioration are the relative stable γ̅ values for GEN-X*n* with decreasing OBS size which results in an overall reduction of auxiliary basis functions. Consequently GEN-X*n* ABS are significantly more efficient than GEN-An** ones preserving similar accuracies. This is particularly obvious in HF calculations.

**Table 2:** Total SCF Energy Variational Density Fitting Errors [kcal/mol] for Various Orbital Basis Sets and Auxiliary Basis Sets in PBE, HF and PBE0 Calculations of the T186 Test Set^a^

basis set	auxis set	γ̅	PBE		HF		PBEO	
			MSE	MAE	MSE	MAE	MSE	MAE
def2-TZVPP	GEN-X2	5.8	–0.04	0.04	4.08	4.08	0.98	0.99
def2-TZVPP	GEN-X3	6.3	–0.03	0.03	0.36	0.36	0.07	0.08
def2-TZVPP	GEN-X4	8.5	–0.02	0.02	0.04	0.04	–0.01	0.02
def2-TZVPP	GEN-A2*	3.6	–0.20	0.20	257.57	257.57	64.54	64.55
def2-TZVPP	GEN-A3*	4.1	–0.07	0.07	93.25	93.25	23.26	23.27
def2-TZVPP	GEN-A4*	6.7	–0.03	0.03	21.31	21.31	5.27	5.28
def2-TZVPP	GEN-A2**	9.4	–0.08	0.08	9.25	9.25	2.25	2.25
def2-TZVPP	GEN-A3**	10.6	–0.03	0.03	0.44	0.44	0.09	0.10
def2-TZVPP	GEN-A4**	17.7	–0.02	0.02	0.02	0.02	–0.01	0.02
6-31G**	GEN-X2	5.6	–0.13	0.13	7.51	7.51	1.76	1.78
6-31G**	GEN-X3	6.2	–0.08	0.08	1.03	1.04	0.19	0.23
6-31G**	GEN-X4	8.6	–0.07	0.07	0.37	0.38	0.04	0.08
6-31G**	GEN-A2*	5.2	–0.41	0.41	326.00	326.01	81.58	81.69
6-31G**	GEN-A3*	7.0	–0.09	0.09	92.78	92.78	23.22	23.22
6-31G**	GEN-A4*	10.6	–0.04	0.04	46.97	46.97	11.70	11.72
6-31G**	GEN-A2**	13.7	–0.17	0.17	26.77	26.77	6.57	6.60
6-31G**	GEN-A3**	18.5	–0.05	0.05	1.66	1.66	0.39	0.40
6-31G**	GEN-A4**	27.7	–0.03	0.03	0.12	0.12	0.01	0.04
DZVP	GEN-X2	6.4	–0.11	0.11	8.82	8.82	2.14	2.15
DZVP	GEN-X3	7.3	–0.06	0.06	1.12	1.13	0.23	0.25
DZVP	GEN-X4	10.0	–0.05	0.05	0.37	0.37	0.05	0.07
DZVP	GEN-A2*	6.5	–0.23	0.23	328.36	328.36	81.79	81.79
DZVP	GEN-A3*	8.5	–0.06	0.06	121.86	121.86	30.33	30.34
DZVP	GEN-A4*	14.4	–0.03	0.03	51.00	51.00	12.67	12.68
DZVP	GEN-A2**	17.0	–0.10	0.10	19.01	19.01	4.67	4.67
DZVP	GEN-A3**	22.4	–0.03	0.03	2.08	2.08	0.49	0.51
DZVP	GEN-A4**	38.0	–0.02	0.02	0.10	0.10	0.00	0.03

a See text for further details.

**Figure 5:**
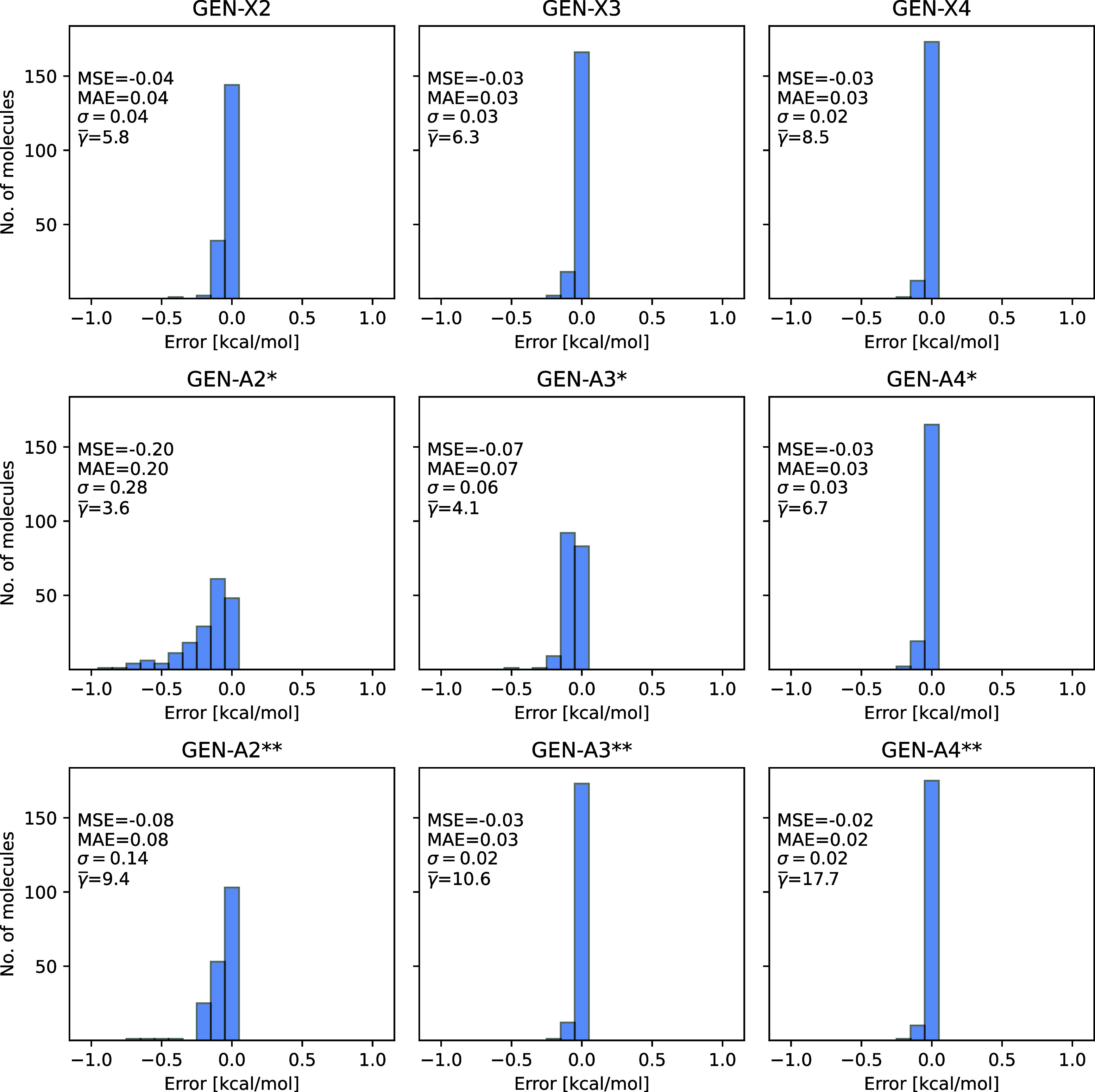
Converged SCF energy errors [kcal/mol] between 3c-ERI and 4c-ERI PBE/def2-TZVPP calculations for the T186 test set molecules. The GEN-X*n*, GEN-An* and GEN-An** (*n* = 2, 3 and 4) auxiliary basis sets are used.

**Figure 6:**
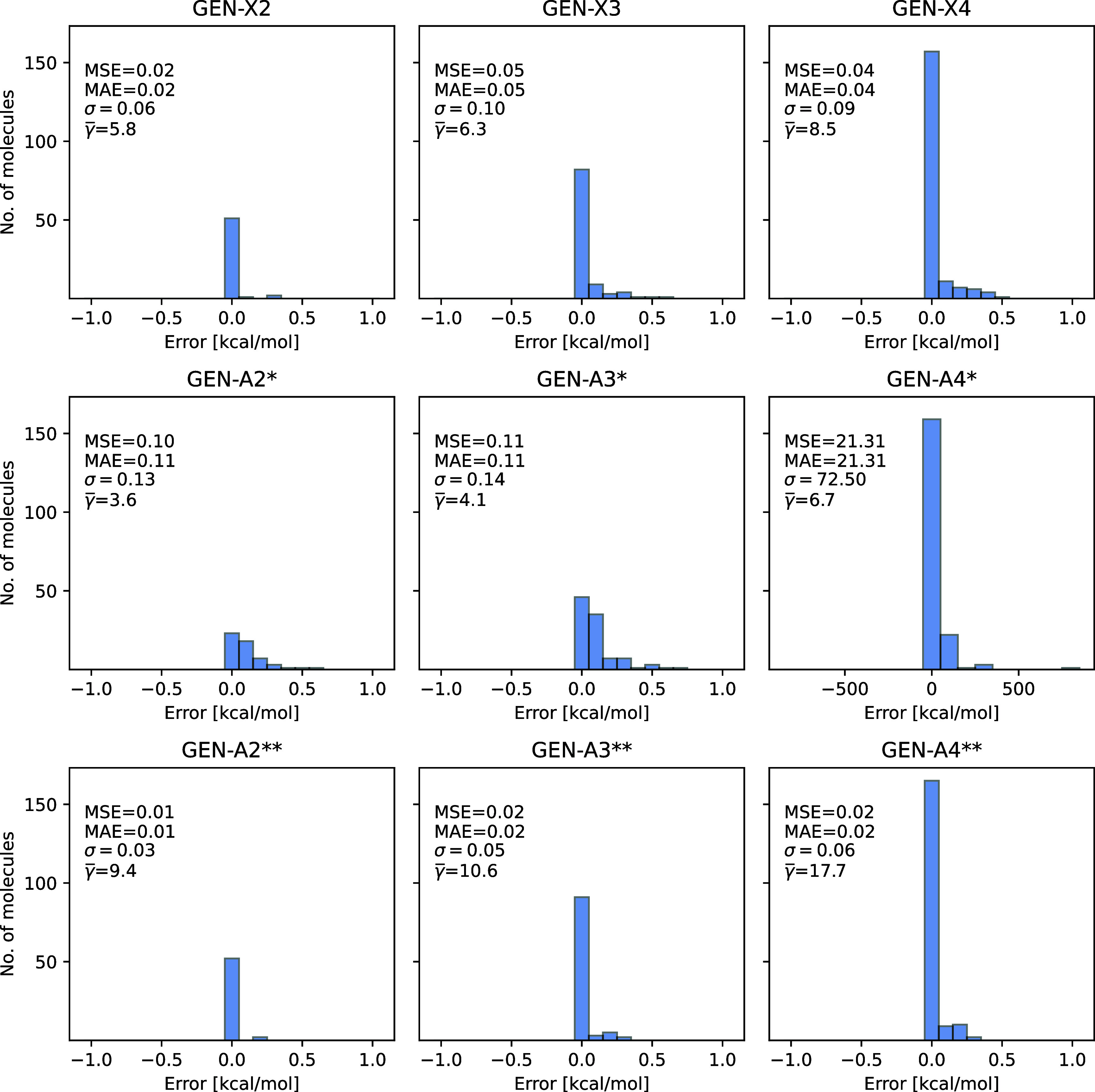
Converged SCF energy errors [kcal/mol] between 3c-ERI and 4c-ERI HF/def2-TZVPP calculations for the T186 test set molecules. The GEN-X*n*, GEN-An* and GEN-An** (*n* = 2, 3 and 4) auxiliary basis sets are used. The left panels (for *n* = 2) include only molecules with elements from H to Ne, the middle panels (for *n* = 3) include molecules with elements from H to Ar and the right panels (for *n* = 4) include all molecules of the T186 test set.

**Figure 7:**
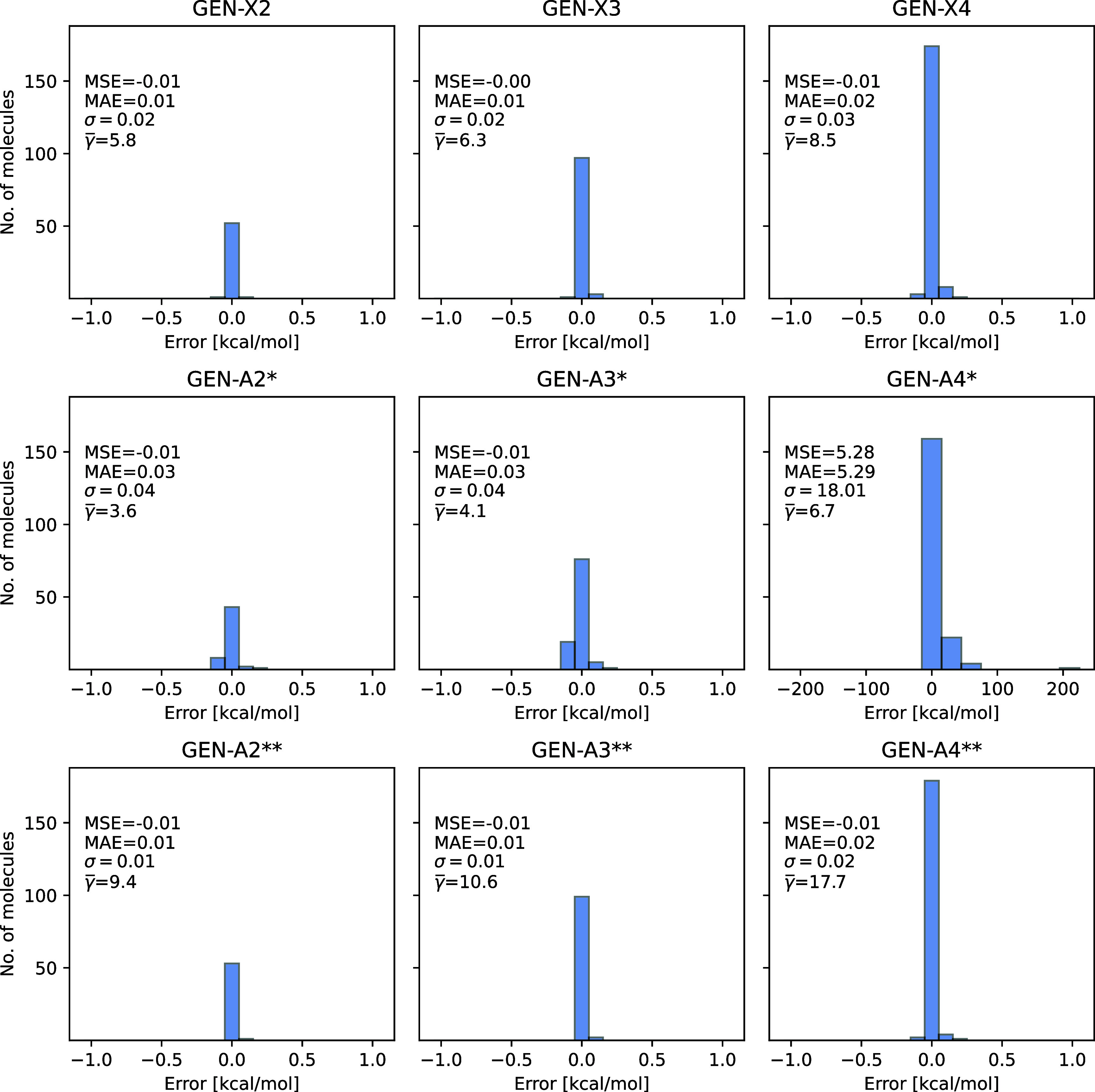
Converged SCF energy errors [kcal/mol] between 3c-ERI and 4c-ERI PBE0/def2-TZVPP calculations for the T186 test set molecules. The GEN-X*n*, GEN-An* and GEN-An** (*n* = 2, 3 and 4) auxiliary basis sets are used. The left panels (for *n* = 2) include only molecules with elements from H to Ne, the middle panels (for *n* = 3) include molecules with elements from H to Ar and the right panels (for *n* = 4) include all molecules of the T186 test set.

Error distribution histograms including all molecules of the T186 test set for the PBE/def2-TZVPP method are depicted in Figure 5. The depicted errors are given by the converged SCF energy difference between the 3c-ERI and 4c-ERI calculations. The panel legend list, besides the γ̅, MSE and MAE from Table 2, also the standard deviation (σ). As Figure 5 shows the errors for all molecules are below 1 kcal/mol and are always negative because of the variational density fitting of the Coulomb energy. Note that this holds for all ABS displayed in Figure 5. The corresponding error distribution diagrams for HF/def2-TZVPP are displayed in Figure 6, where all errors (within a resolution of 0.05 kcal/mol) are positive due to the larger (positive) error of the variational fitted Fock exchange energy with respect to its Coulomb counterpart. Because of fitting error increase in the Fock exchange energy for elements with higher atomic numbers, we separated the T186 test set into three parts: The left panels in Figure 6 (*n* = 2) include only molecules of the T186 test set with elements from H to Ne, the middle panels (*n* = 3) include molecules of this test set with elements from H to Ar and the right panels (*n* = 4) of this figure include all molecules of this test set. As Figure 6 shows the GEN-An* ABS yield fitting precisions below 1 kcal/mol for molecules consisting of light elements (H to Ar) but fail dramatically for heavier element molecules (right panel in the middle row of Figure 6). This failure can be rectified with the GEN-A4** ABS which is, however, much too large  for large scale applications. The newly developed GEN-X4 ABS (top row right panel in Figure 6) closes this gap by delivering GEN-A4** accuracy for molecules with heavy elements with a much reduced number of auxiliary basis functions . The error distribution diagrams for the PBE0/def2-TZVPP method are displayed in Figure 7. Here positive and negative errors occur due to the reduced amount of Fock exchange in hybrid functionals. The overall behavior is similar to the HF diagrams in Figure 6. Therefore, GEN-X4 is also an attractive ABS for density fitted hybrid Kohn–Sham DFT calculations if heavy elements are involved. The five largest MSE for all three comparisons are given in Table S1 of the Supporting Information.

### 4.2 G3 Enthalpies of Formation

**Figure 8:**
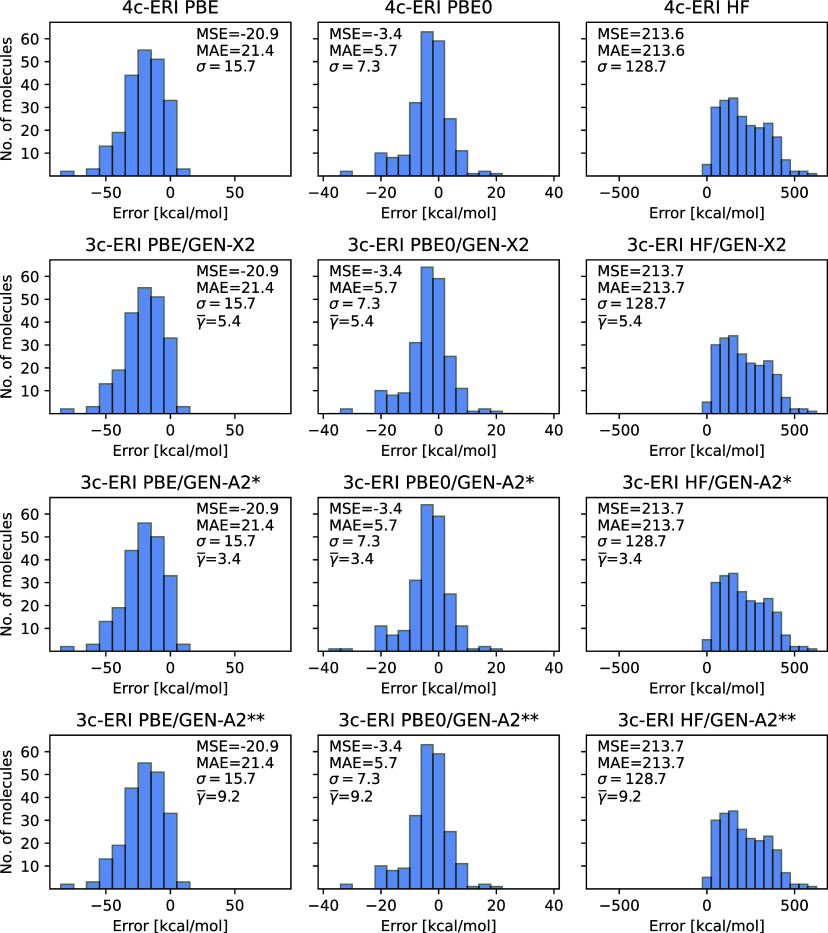
Formation enthalpy errors [kcal/mol] of PBE, PBE0 and HF calculations employing the def2-TZVPP orbital basis set for the G3 test set. First row results are from 4c-ERI Psi4 calculations and the following rows show 3c-ERI deMon2k results with the GEN-X2, GEN-A2* and GEN-A2** auxiliary basis sets, respectively.

The accuracy of the new ABS GEN-X2 for the calculation of formation enthalpies is studied with the G3 test set as outlined in the Computational Methods. For comparison, the formation enthalpies are calculated with the GEN-A2* and GEN-A2** ABS, too. Figure 8 depicts error distribution histograms with respect to experiment for the PBE, PBE0 and HF methods, employing the def2-TZVPP OBS together with the GEN-X2, GEN-A2* and GEN-A2** ABS. As Figure 8 shows all 3c-ERI Kohn–Sham (PBE and PBE0) formation enthalpy errors are identical to their 4c-ERI counterparts. For HF a small difference of 0.1 kcal/mol in the MSE and MAE are found between the 4c-ERI and 3c-ERI formation enthalpy errors. This underlines the quality of the GEN-X2, GEN-A2* and GEN-A2** ABS for molecules containing first and second row atoms. We note that the excellent performance for the calculation of formation enthalpies of the here discussed ABS also translates to other chemical relevant energies such as activation and reaction energies.

**Figure 9:**
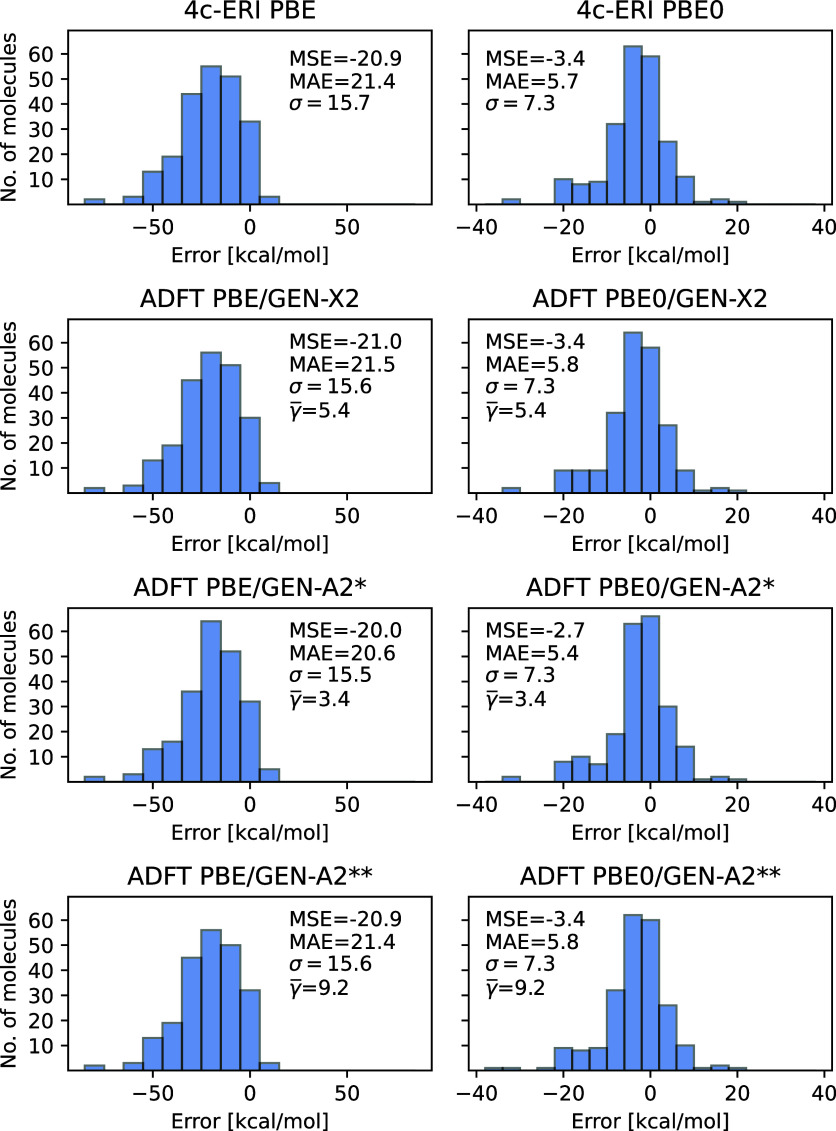
Formation enthalpy errors [kcal/mol] of ADFT PBE and ADFT PBE0 calculations employing the def2-TZVPP orbital basis set for the G3 test set. For comparison the 4c-ERI Psi4 enthalpy errors are given in the first row, too. The following rows show 3c-ERI deMon2k ADFT results with the GEN-X2, GEN-A2* and GEN-A2** auxiliary basis sets, respectively.

Besides Kohn–Sham (VXCTYPE BASIS) energies deMon2k permits the calculation of ADFT (VXCTYPE AUXIS) energies, too. Because the exchange–correlation energy and potential is calculated with the auxiliary density in ADFT the energy bounds shift. Therefore, a direct comparison of ADFT SCF energies with corresponding 4c-ERI Kohn–Sham results is meaningless. Nevertheless, energy differences and thus formation enthalpies can be compared. Figure 9 depicts ADFT formation enthalpy error distribution histograms with respect to experiment for the PBE and PBE0 methods employing the def2-TZVPP OBS in combination with the GEN-X2, GEN-A2* and GEN-A2** ABS. As the error statistics in Figure 9 shows ADFT PBE and PBE0 calculations with the GEN-X2 and GEN-A2** ABS reproduce the 4c-ERI formation enthalpy errors within 0.1 kcal/mol. Note that GEN-X2 with γ̅ = 5.4 is computationally more efficient than GEN-A2** with γ̅ = 9.2. The corresponding GEN-A2* ADFT formation enthalpy errors are still in fair agreement with their 4c-ERI counterparts but show with up to 0.9 kcal/mol a significantly larger discrepancy than the GEN-X2 and GEN-A2** results. As for the Kohn–Sham calculations the excellent performance of the GEN-X2 and GEN-A2** ABS for ADFT formation enthalpies translates to other chemically relevant energies, too.

### 4.3 Benchmark Calculations

**Figure 10:**
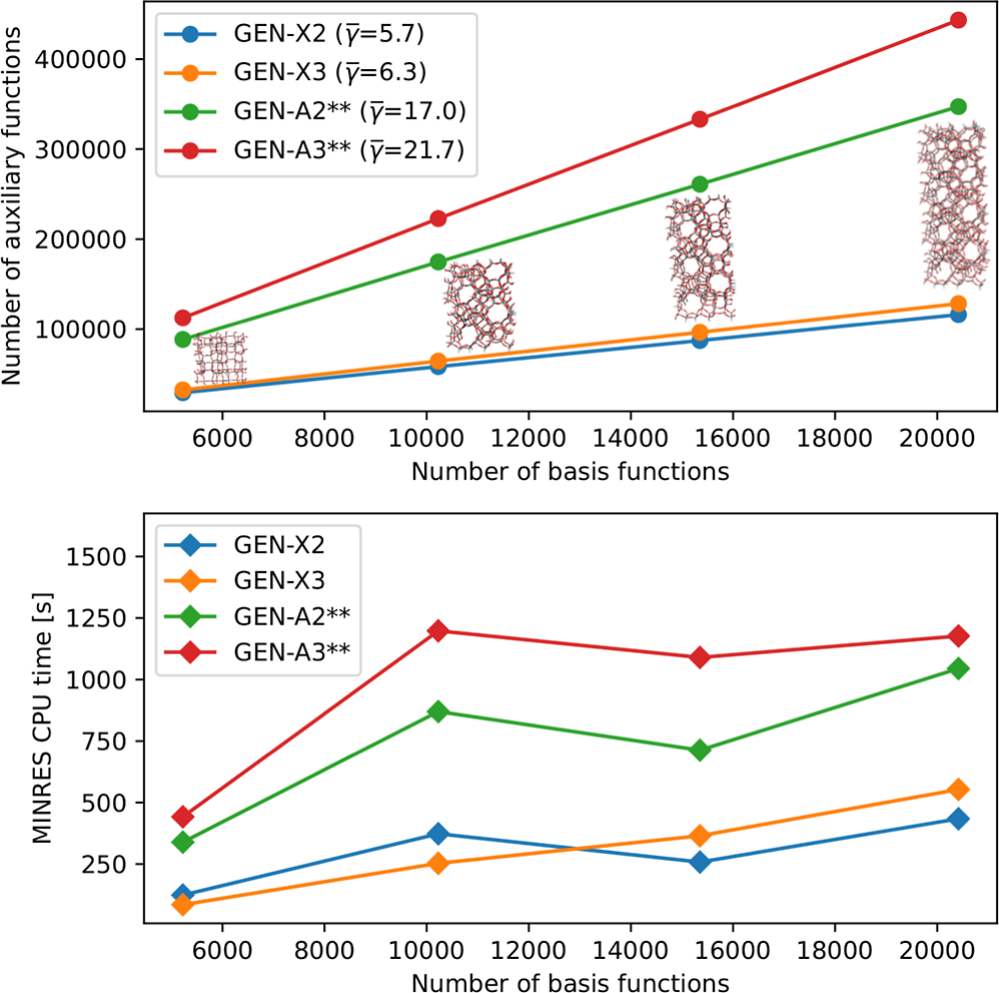
Number of auxiliary basis functions vs orbital basis functions for hydrogen saturated MFI zeolites cutouts with 376, 720, 1064, and 1408 atoms in the top panel. MINRES density fitting CPU times [s] per SCF cycle vs orbital basis functions in the lower panel.

Most parts of variational density-fitting calculations scale with the number of auxiliary basis functions. Thus, ABS with small γ̅ are usually computationally most efficient. An exception is the solution of eq 12 with the MINRES approach. Here the number of auxiliary basis functions competes with the number of MINRES iterations. To benchmark the MINRES fitting for the GEN-X*n* ABS we performed single-point energy calculations of hydrogen saturated MFI zeolites at the ADFT PBE/DZVP level of theory with the GEN-X2 and GEN-X3 ABS. For comparison, we also performed these calculations with the GEN-A2** and GEN-A3** ABS. Figure 10 depicts the relation between the number of orbital basis and auxiliary basis functions in the top panel. As this graph shows the number of auxiliary basis functions generated by the GEN-X*n* algorithm are much smaller than those generated by the GEN-An** ones. The difference is roughly a factor of 4 which is reflected in the corresponding γ̅ values, too. The lower panel of Figure 10 depicts the MINRES density fitting CPU time [s] per SCF cycle as a function of the number of orbital basis functions. As expected the GEN-X*n* sets outperforms the GEN-An** ones due to the reduced number of auxiliary basis functions.

More surprising is the comparison between GEN-X2 and GEN-X3. Although GEN-X2 produces for the MFI cutouts around 10% less auxiliary basis functions than GEN-X3, the GEN-X3 MINRES fitting CPU times for the first two MFI cutouts are smaller than for GEN-X2 (see Figure 10). Somewhere above 10 000 orbital basis functions the timings invert and GEN-X2 becomes faster than GEN-X3. We attribute this behavior to the improved MINRES convergence for GEN-X3 in the smaller MFI cutouts. Note also the linear scaling of the GEN-X3 MINRES CPU time with respect to the number of orbital basis functions. Independent from the performance of the tested ABS these benchmark calculations demonstrate that MINRES density fitting for systems with more than 400 000 auxiliary basis functions is possible on compute architectures with 4 GB random access memory (RAM) per core.

## 5 Conclusions

A new algorithm for the automatic generation of auxiliary basis sets (ABS) with shared exponents is presented. For simplicity and transferability between underlying orbital basis sets (OBS) even-tempered exponents are used. The resulting GEN-X*n* (*n* = 2, 3 and 4) ABS yield fitting precision below 1 kcal/mol in the converged SCF energies for all test systems containing elements from H to Kr for the DZVP, 6-31G** and def2-TZVPP OBS. In all PBE and HF calculations the theoretical error bound signs for the variational density fitting of the two-electron Coulomb and Fock energies are observed. This underlines the accuracy and numerical stability of the variational density fitting for Kohn–Sham and Hartree–Fock calculations. Benchmark results for hydrogen saturated MFI zeolite cutouts reveal the computational efficiency of the GEN-X*n* ABS compared to the GEN-An** ones. Besides the reduction of the number of auxiliary basis functions by almost a factor of 4 without sacrificing accuracy, improved convergence of the MINRES iterative density fitting is observed, too.

The ADFT enthalpy calculations with PBE and PBE0 employing the G3 test set show excellent agreement with corresponding four-center ERI Psi4 calculations when employing the GEN-X2 ABS. This indicates that the GEN-X*n* ABS are not only suitable for Kohn–Sham but also for ADFT calculations. This is particularly beneficial for hybrid functional Born–Oppenheimer molecular dynamics simulations where the computational efficiency of ADFT is mandatory for obtaining statistical meaningful results in reasonable wall clock times. To extend the application range of the GEN-X*n* algorithm to the full periodic table adoption to effective and model core potentials is currently under development in our laboratories.

## Supporting Information Available

The following file is available free of charge. The Supporting Information is available free of charge at https://pubs.acs.org/doi/10.1021/acs.jctc.4c01555.

BAFS.pdf: Figures S1 to S36 are plots of orbital basis sets and auxiliary basis sets for elements from H to Kr. Table S1 lists the five signed largest SCF energy errors for the T186 test set (PDF)
