# Supplementary material for: Automatic Generation of Even-Tempered Auxiliary Basis Sets with Shared Exponents for Density Fitting
Source: J Chem Theory Comput. 2025 Feb 27;21(5):2338–52. doi: 10.1021/acs.jctc.4c01555 (PMC11912211; doi:10.1021/acs.jctc.4c01555)
Supplement: Supplementary file 1 — ct4c01555_si_001.pdf [file ct4c01555_si_001.pdf]

Supporting Information

for

Automatic Generation of Even-Tempered Auxiliary Basis Sets with Shared Exponents for Density Fitting

Manuel Díaz-Tinoco,† Roberto Flores-Moreno,‡ Bernardo A. Zúñiga-Gutiérrez,‡ and Andreas M. Köster*,†

†*Departamento de Química, CINVESTAV, Avenida Instituto Politécnico Nacional 2508 A.P. 14-740 Mexico D.F. 07000, Mexico*

‡*Departamento de Química, Universidad de Guadalajara. Blvd. Marcelino García Barragán 1421, Guadalajara Jalisco, C.P. 44430, Mexico*

E-mail: akoster@cinvestav.mx

Plots of the number of basis and auxiliary functions vs. exponent values for the 6-31G** (left) and def2-TZVPP (right) orbital basis sets for all elements from H to Kr. The automatically generated GEN-Xn, GEN-An* and GEN-An** auxiliary basis sets are shown in the lower panels, $n = 2$ for H to Ne, $n = 3$ for Na to Ar and $n = 4$ for K to Kr.

Figure S1: Plots for H

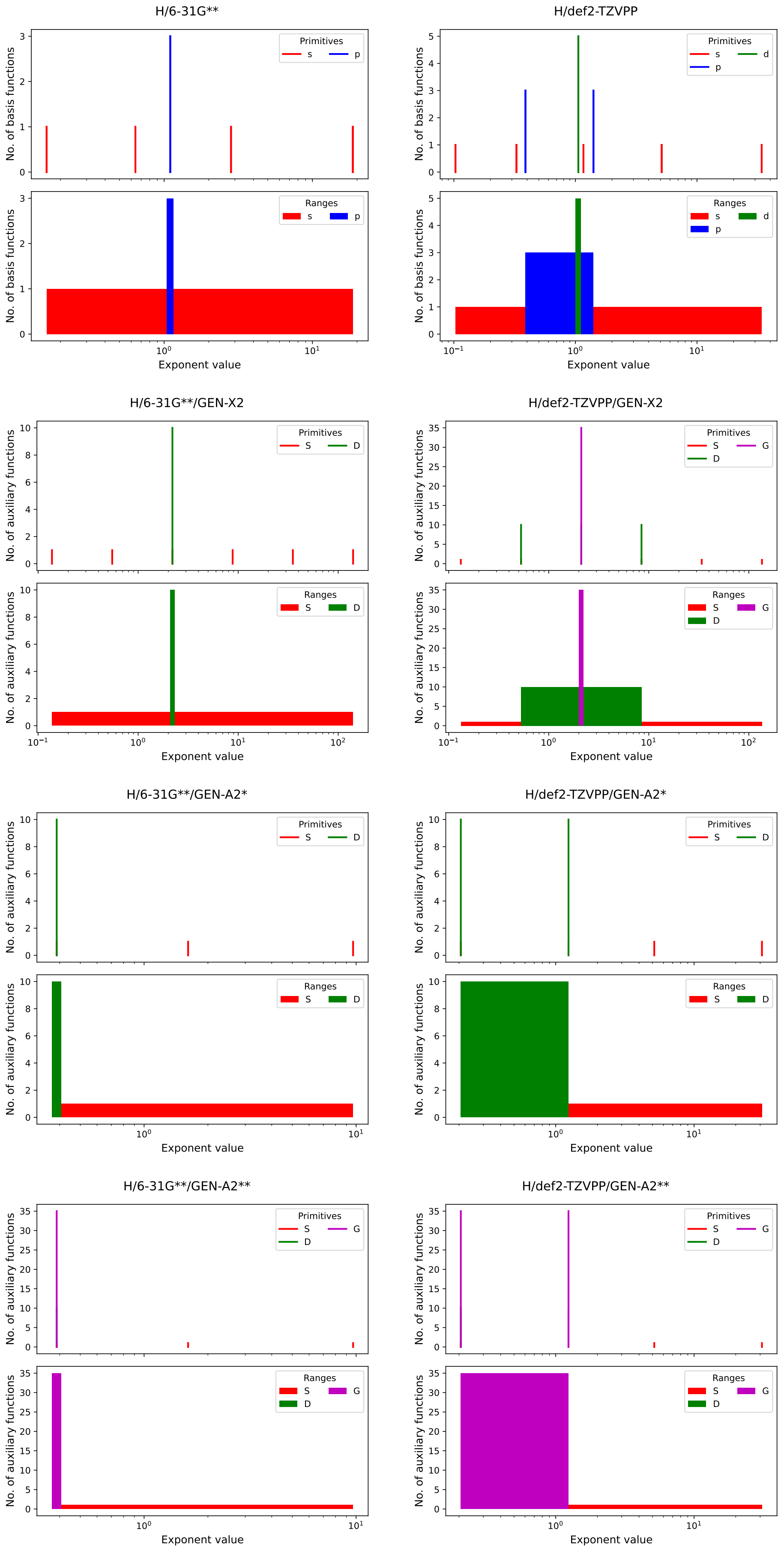

Figure S2: Plots for He

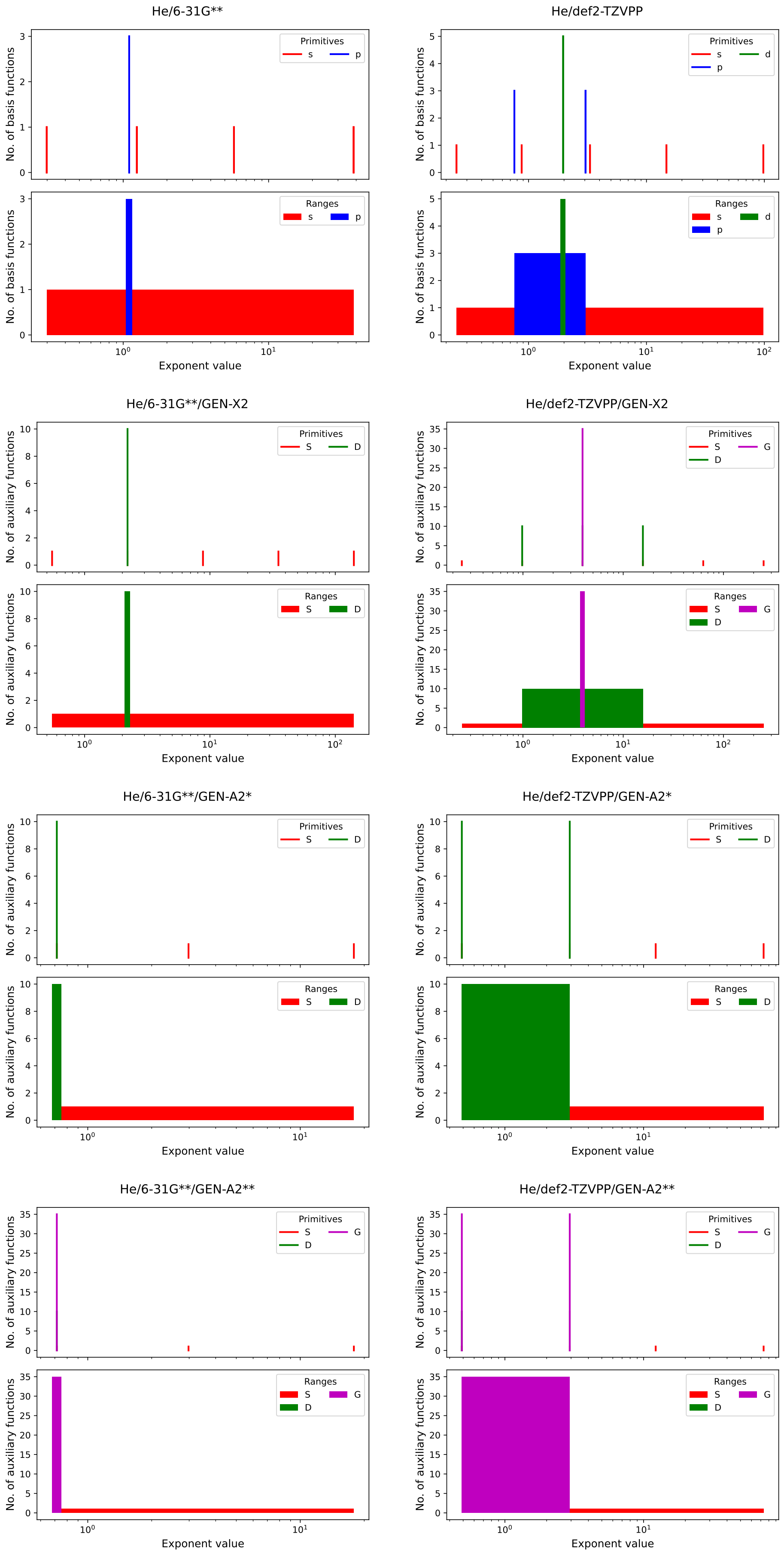

Figure S3: Plots for Li

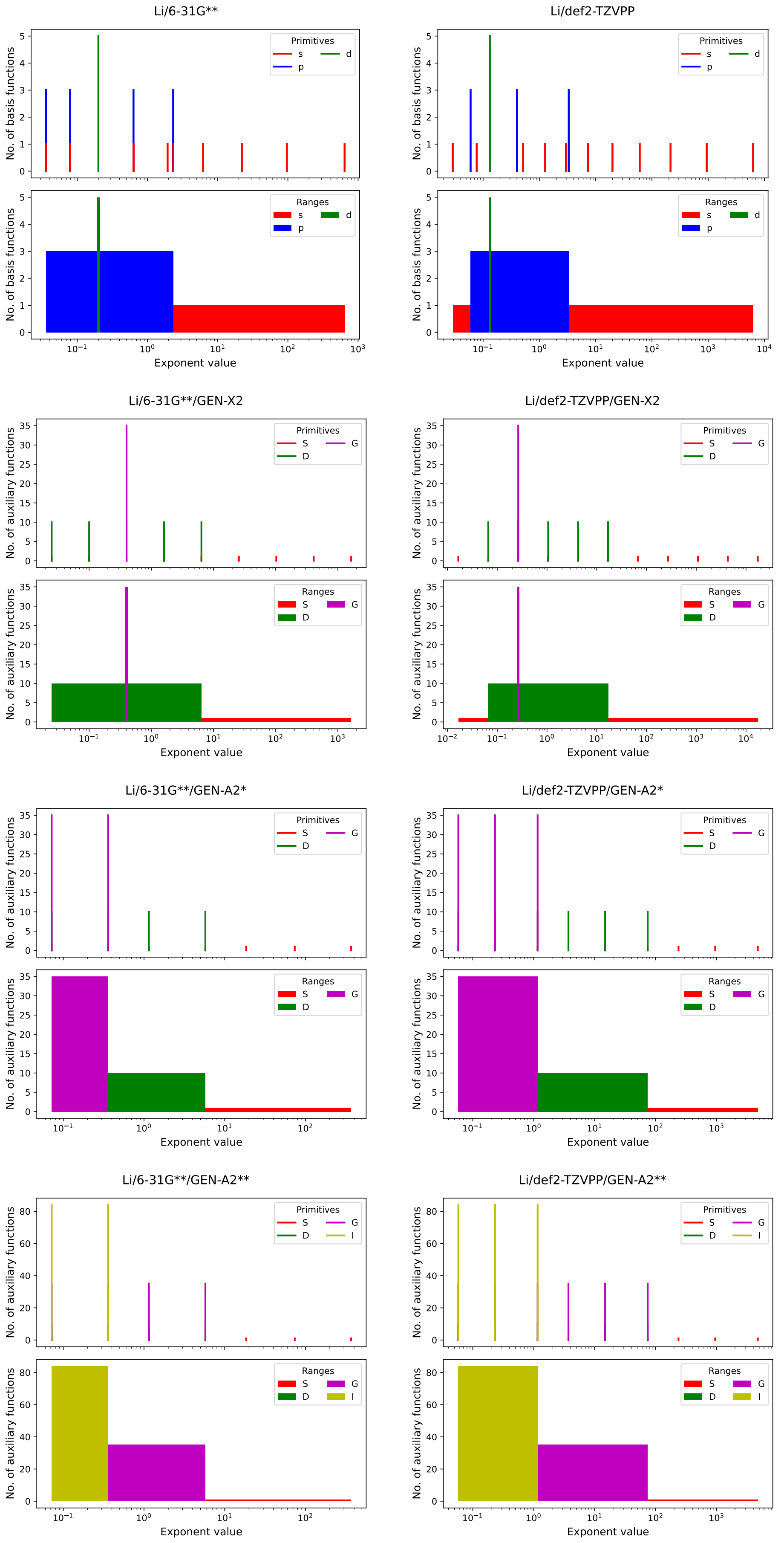

Figure S4: Plots for Be

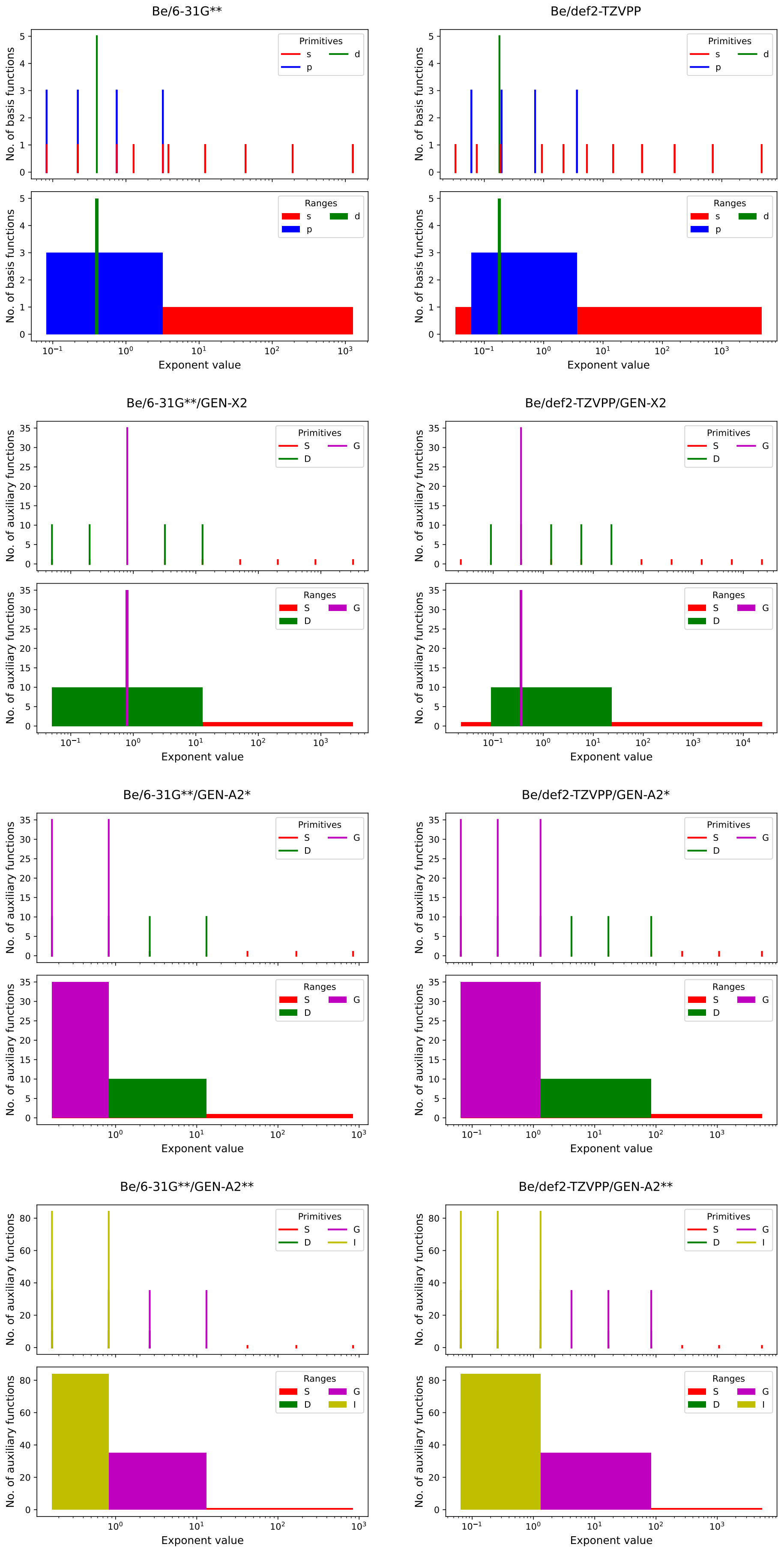

Figure S5: Plots for B

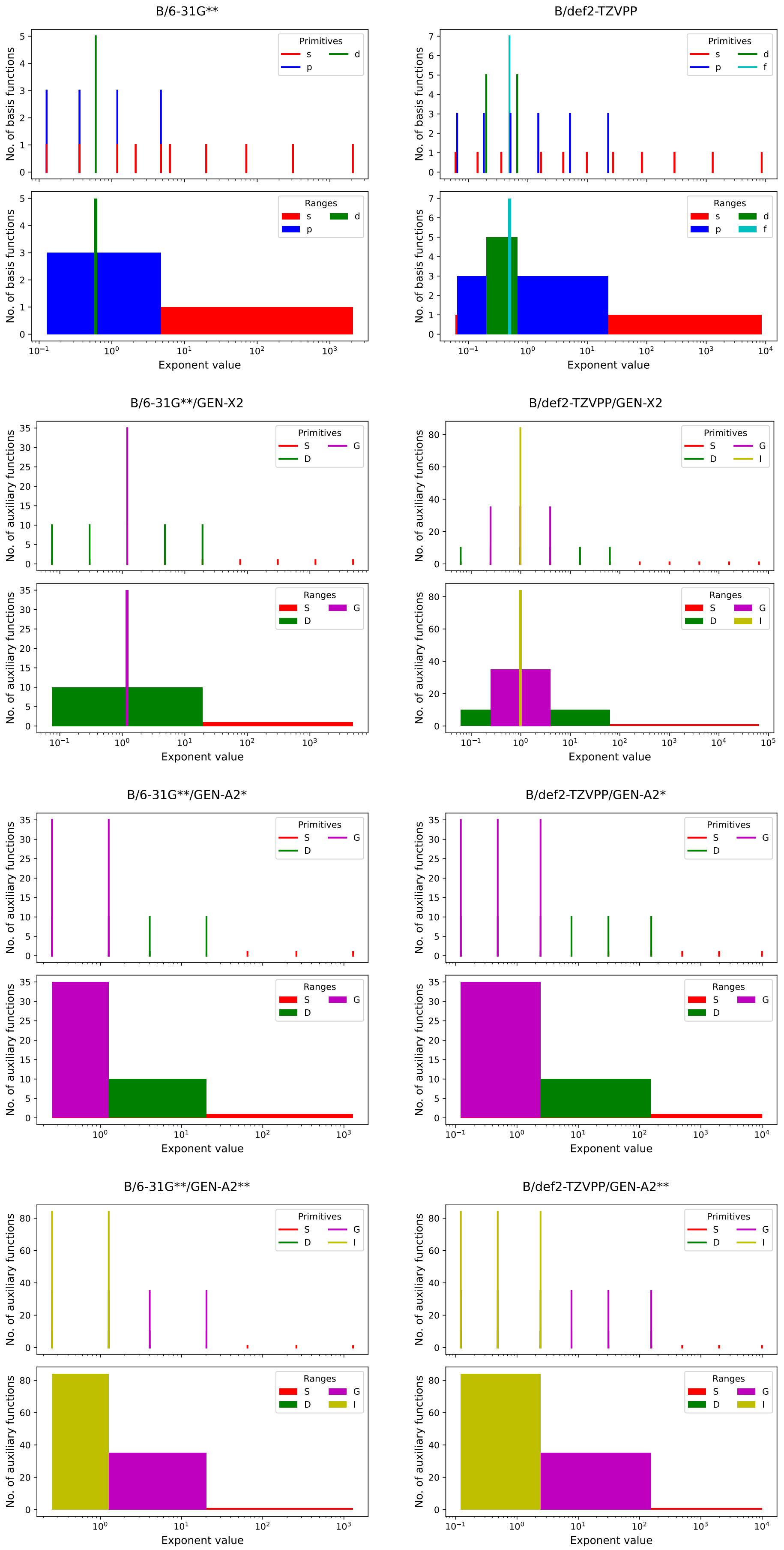

Figure S6: Plots for C

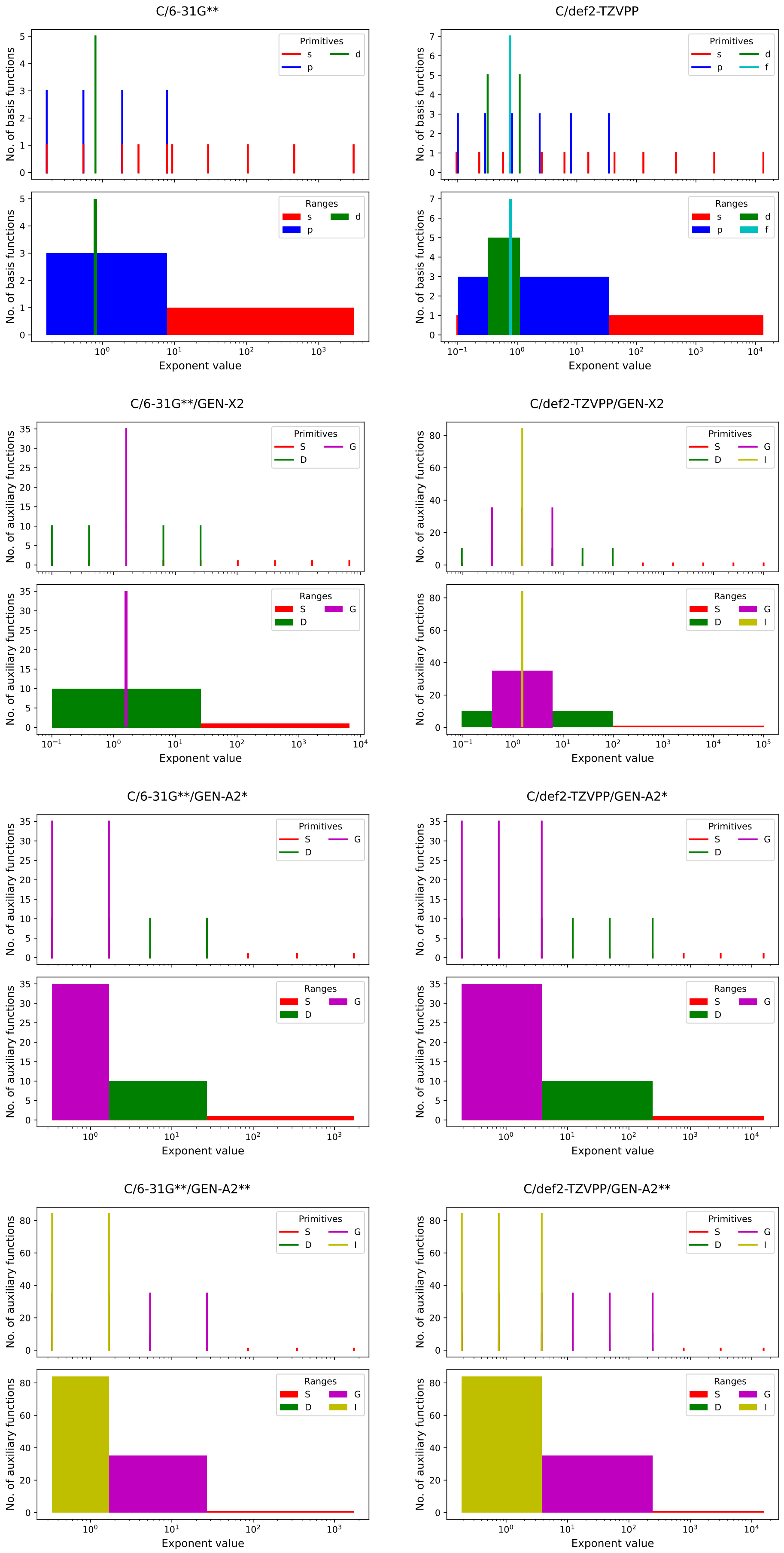

Figure S7: Plots for N

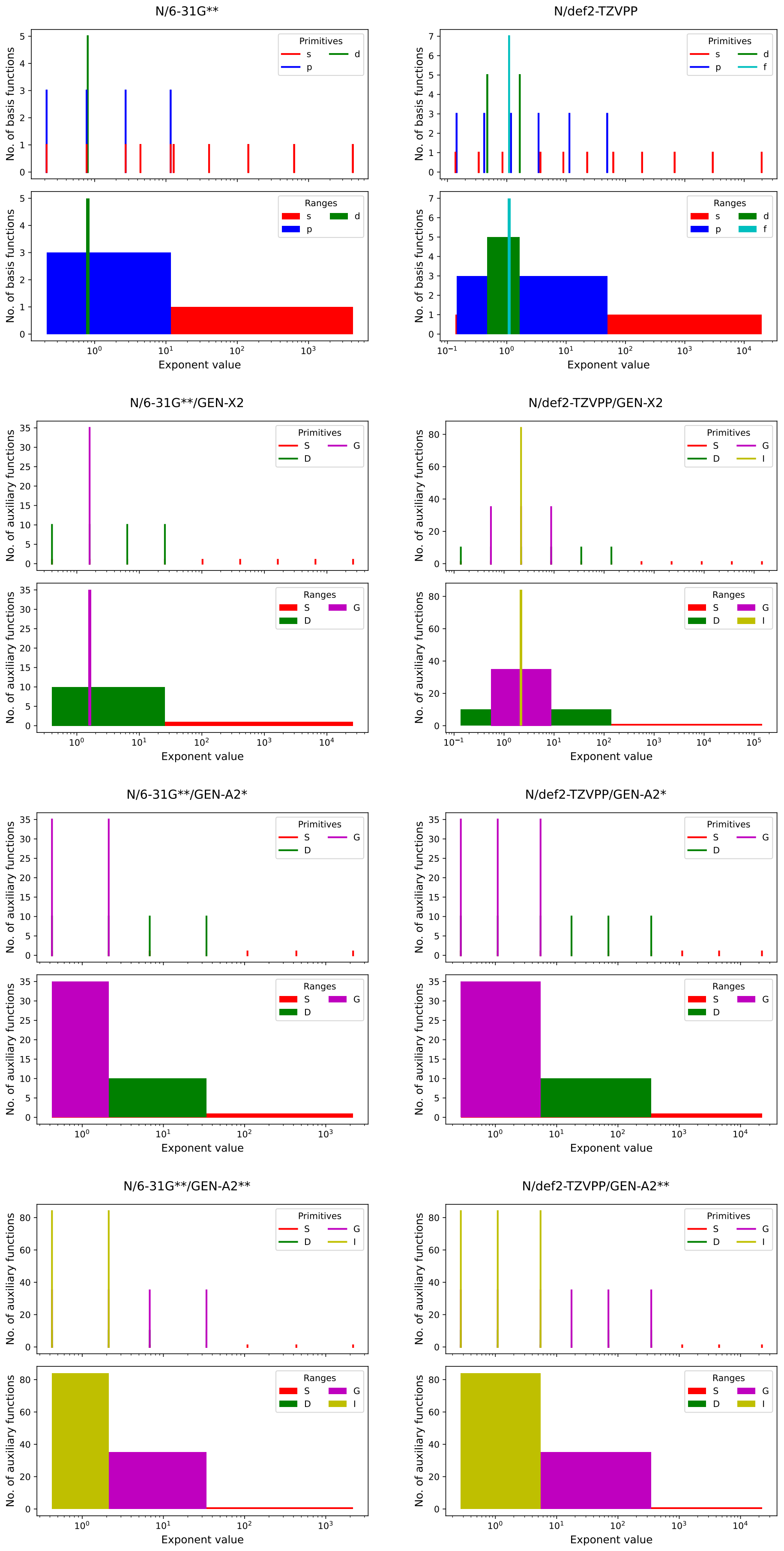

Figure S8: Plots for O

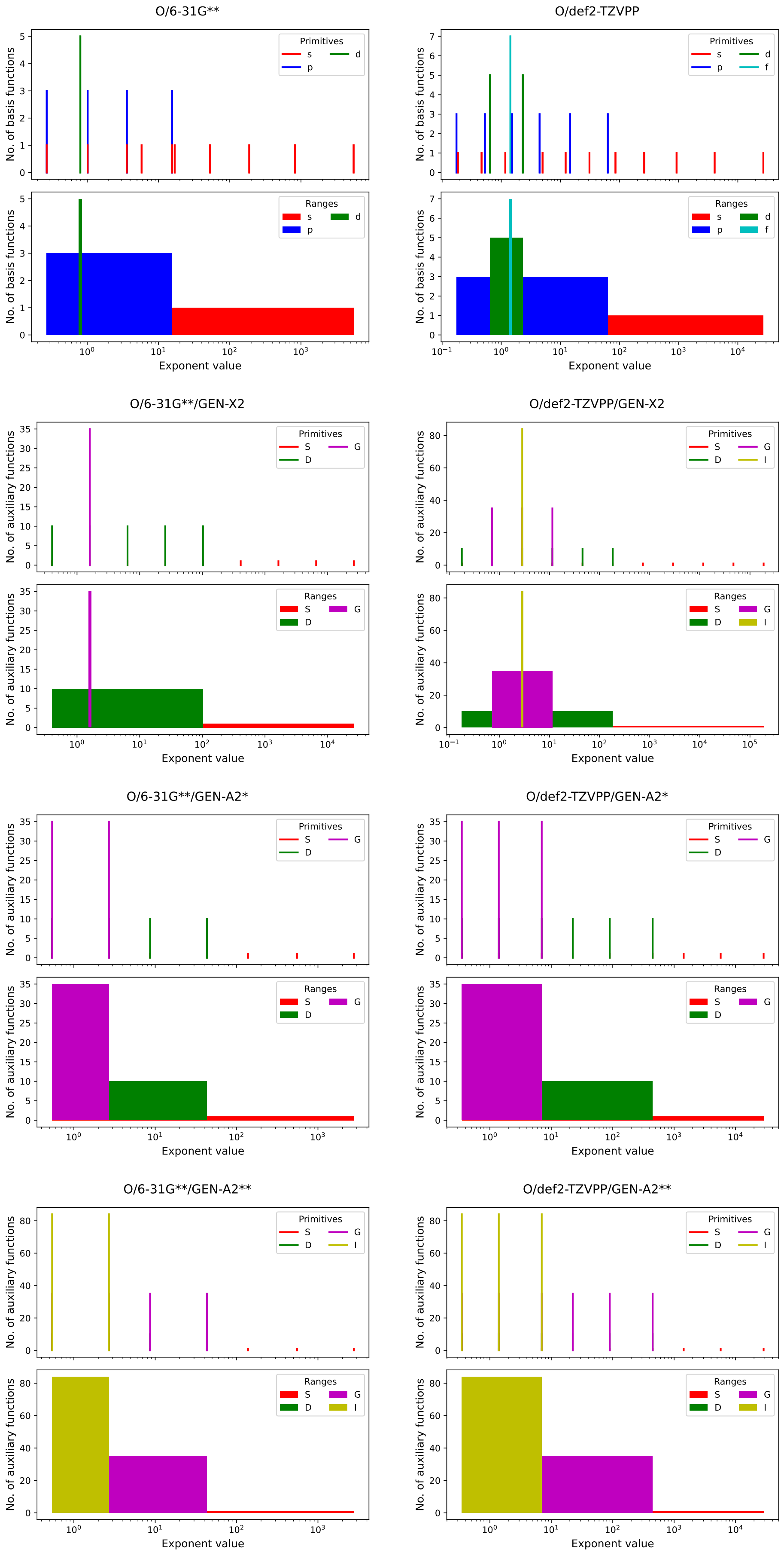

Figure S9: Plots for F

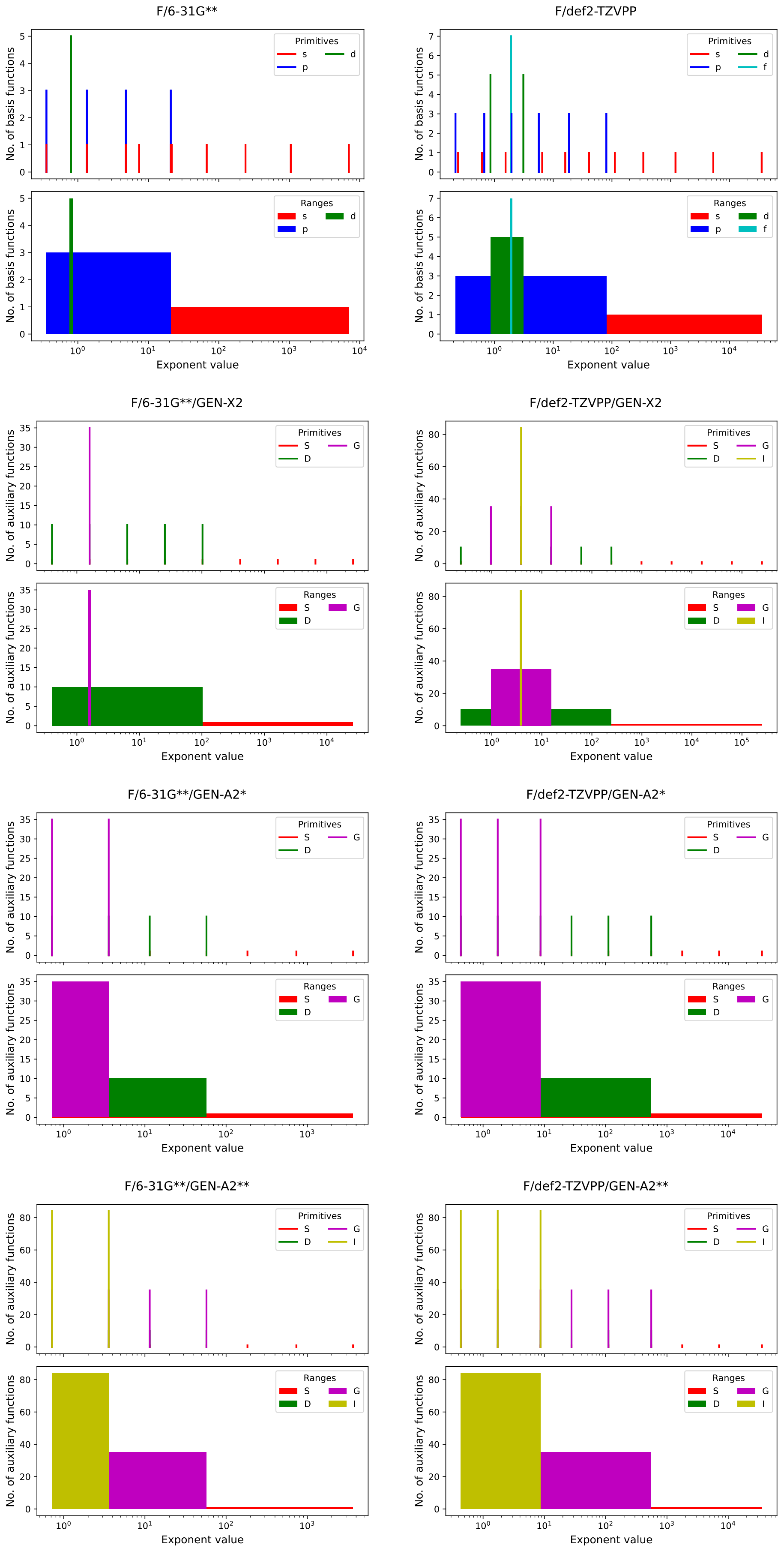

Figure S10: Plots for Ne

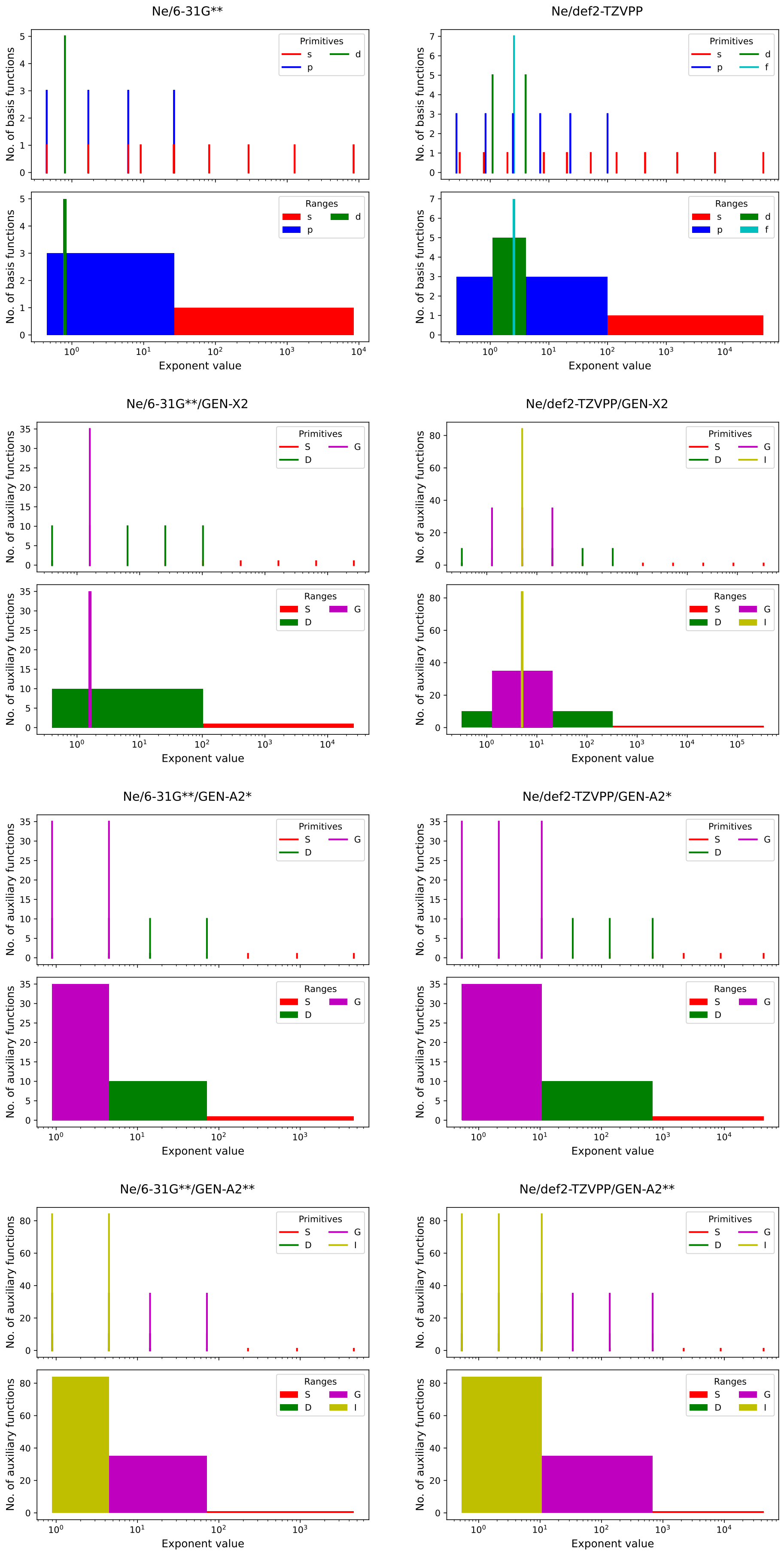

Figure S11: Plots for Na

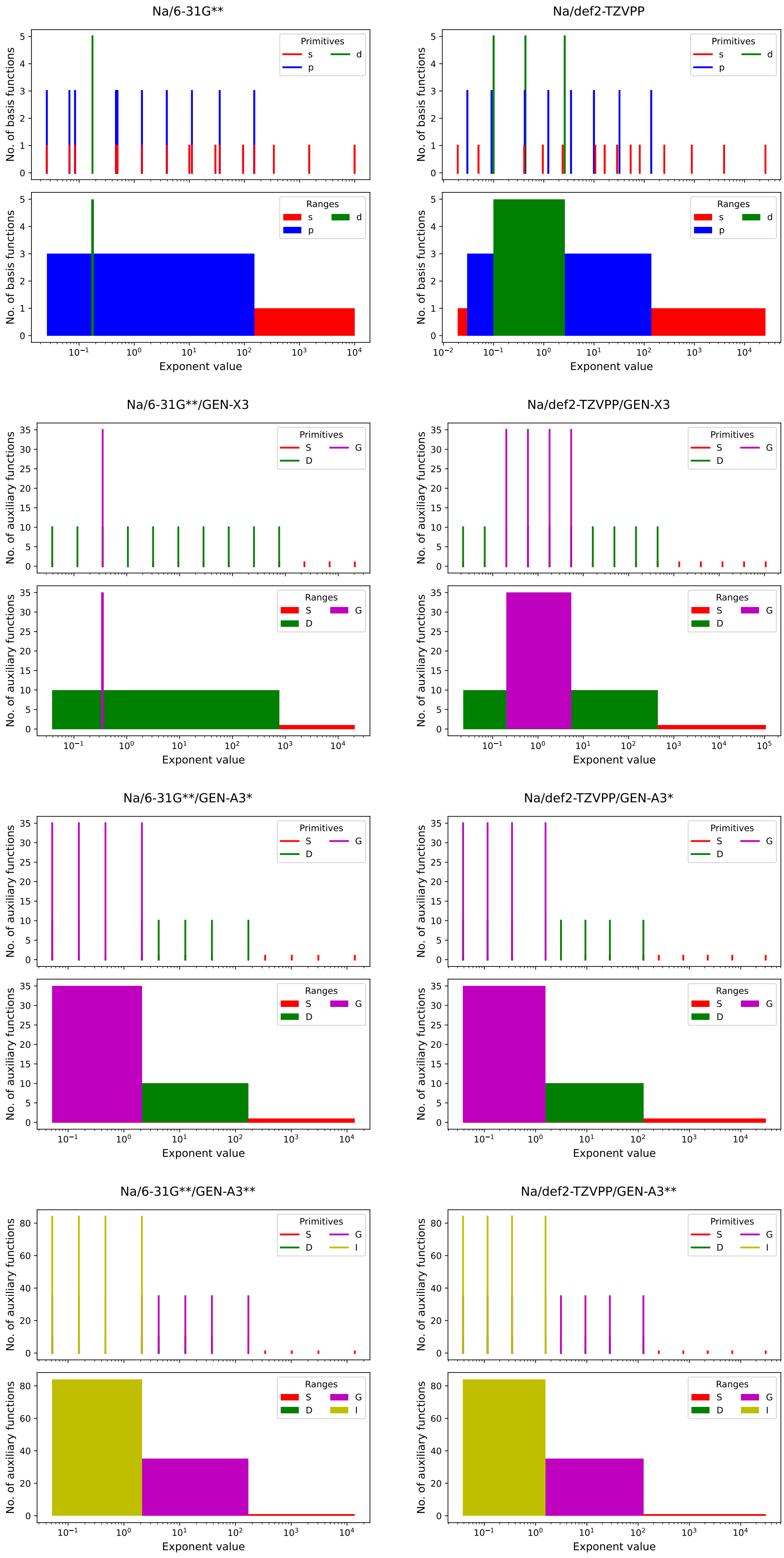

Figure S12: Plots for Mg

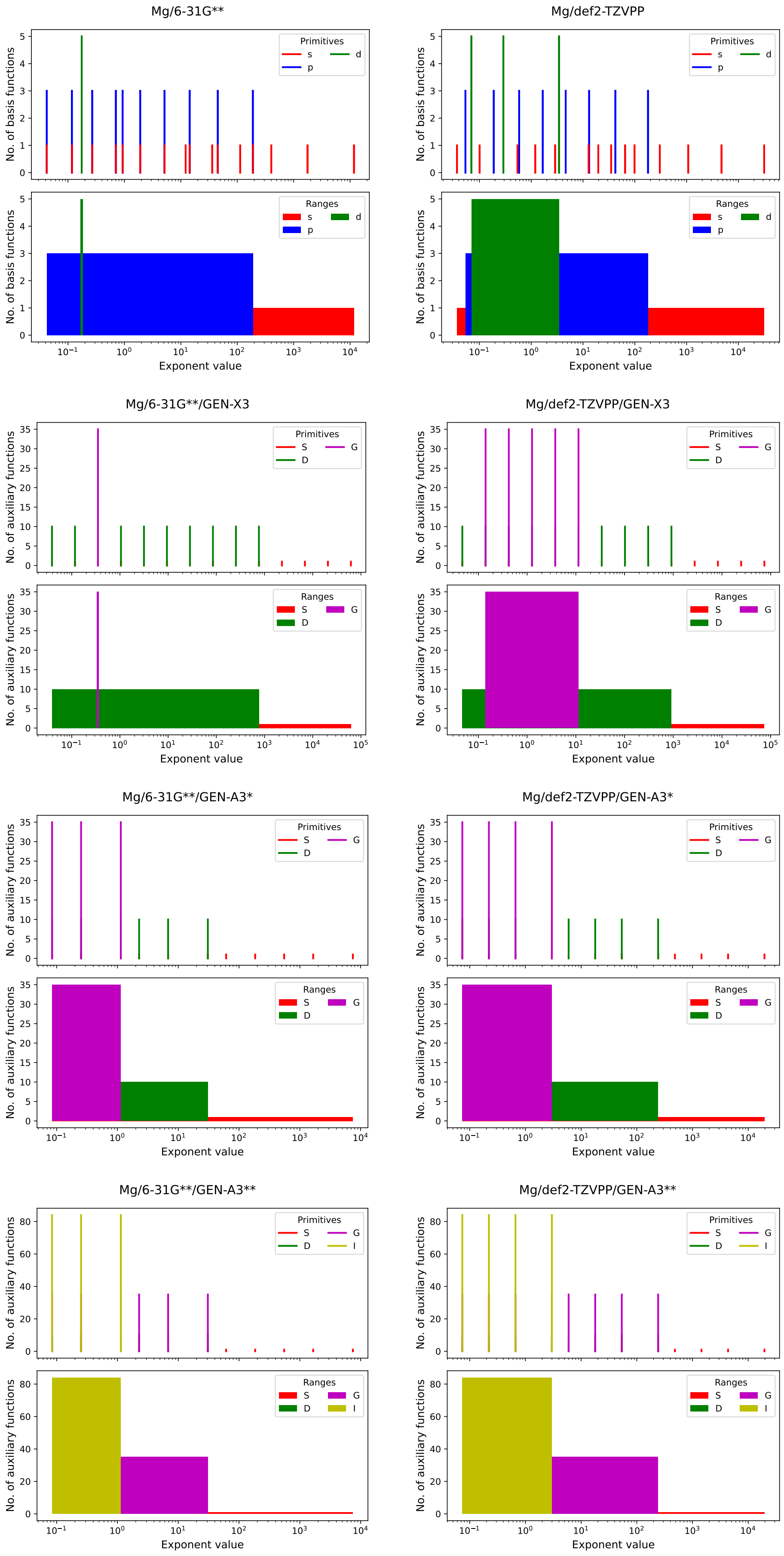

Figure S13: Plots for Al

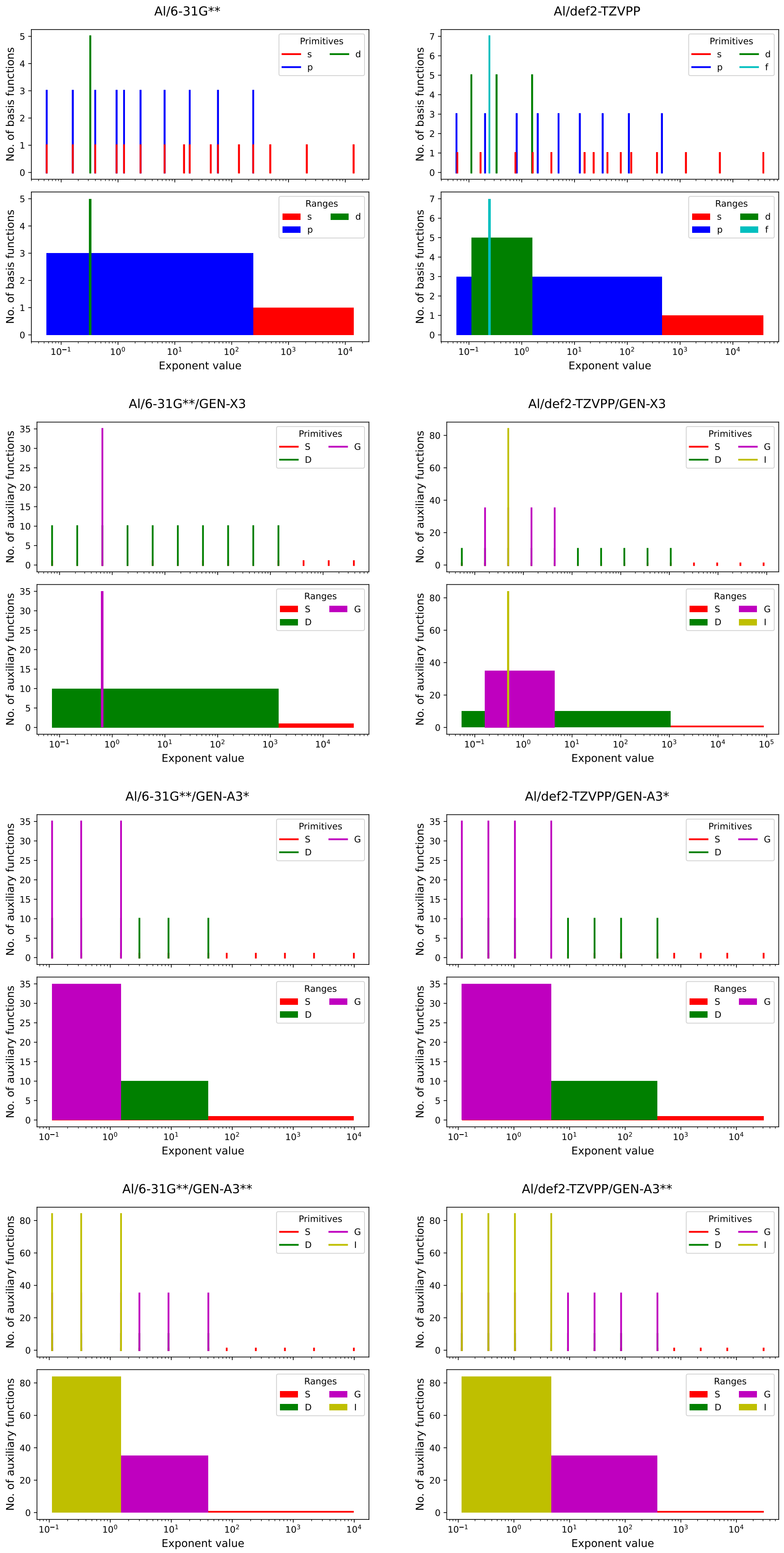

Figure S14: Plots for Si

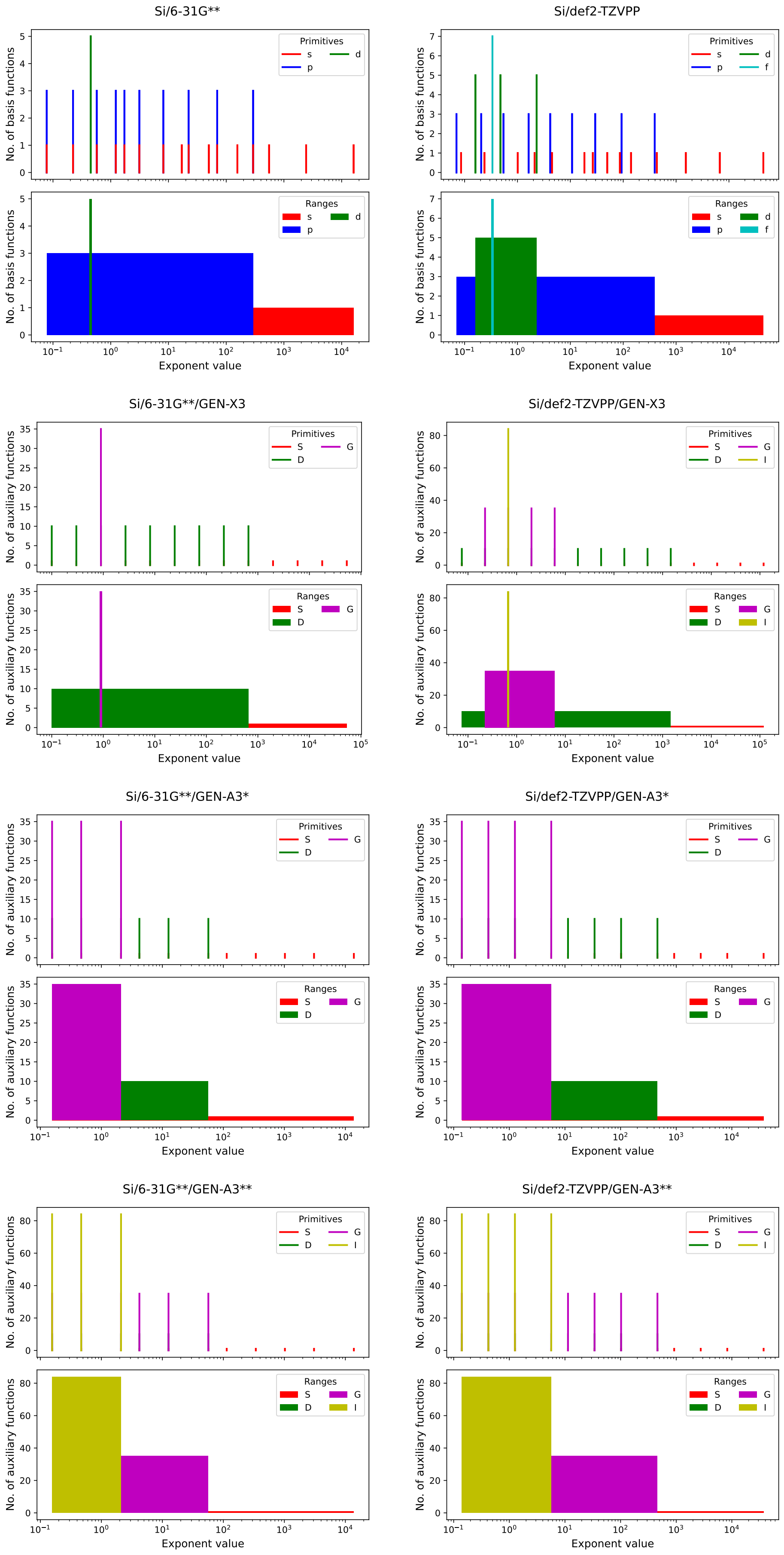

Figure S15: Plots for P

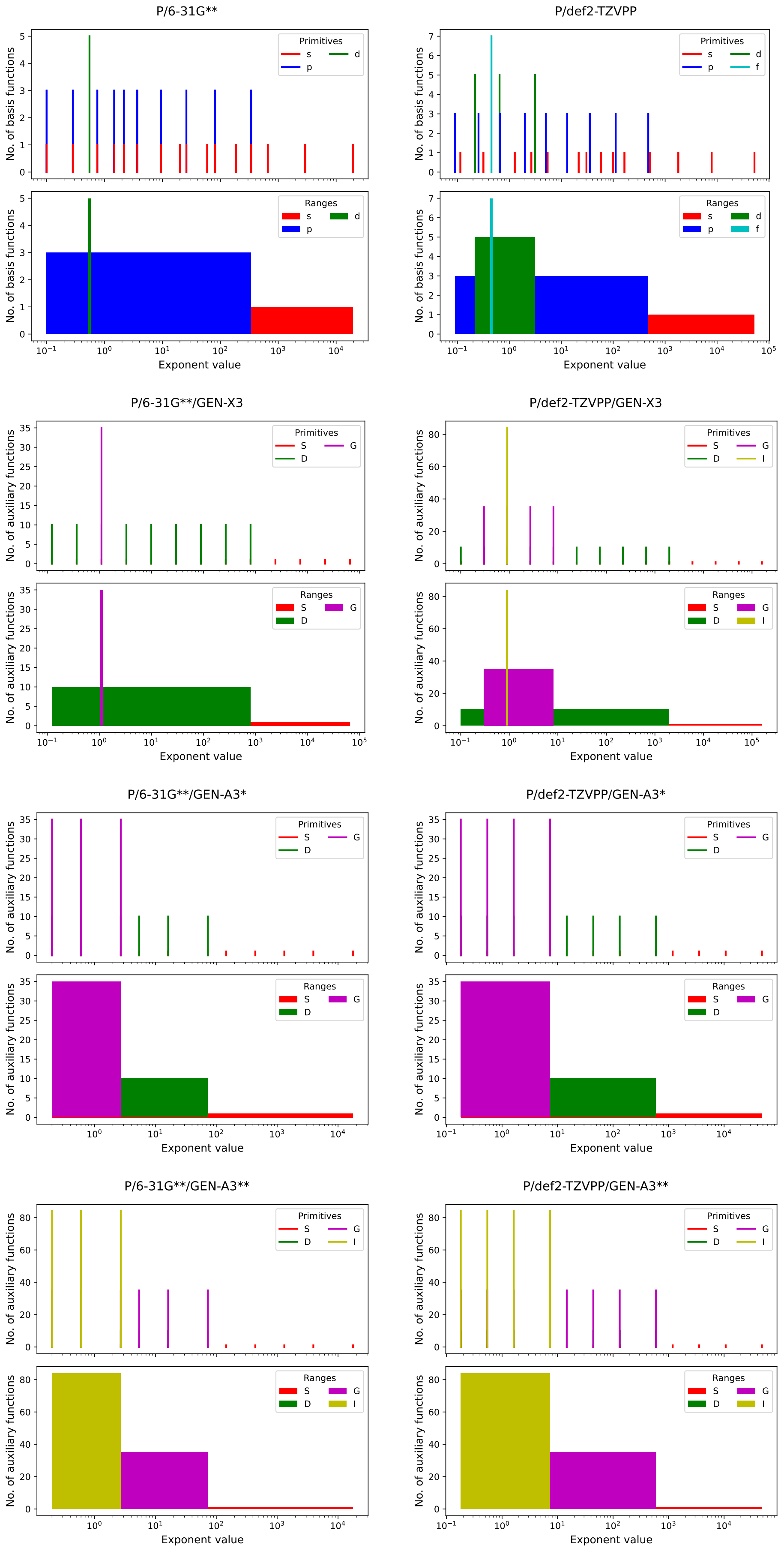

Figure S16: Plots for S

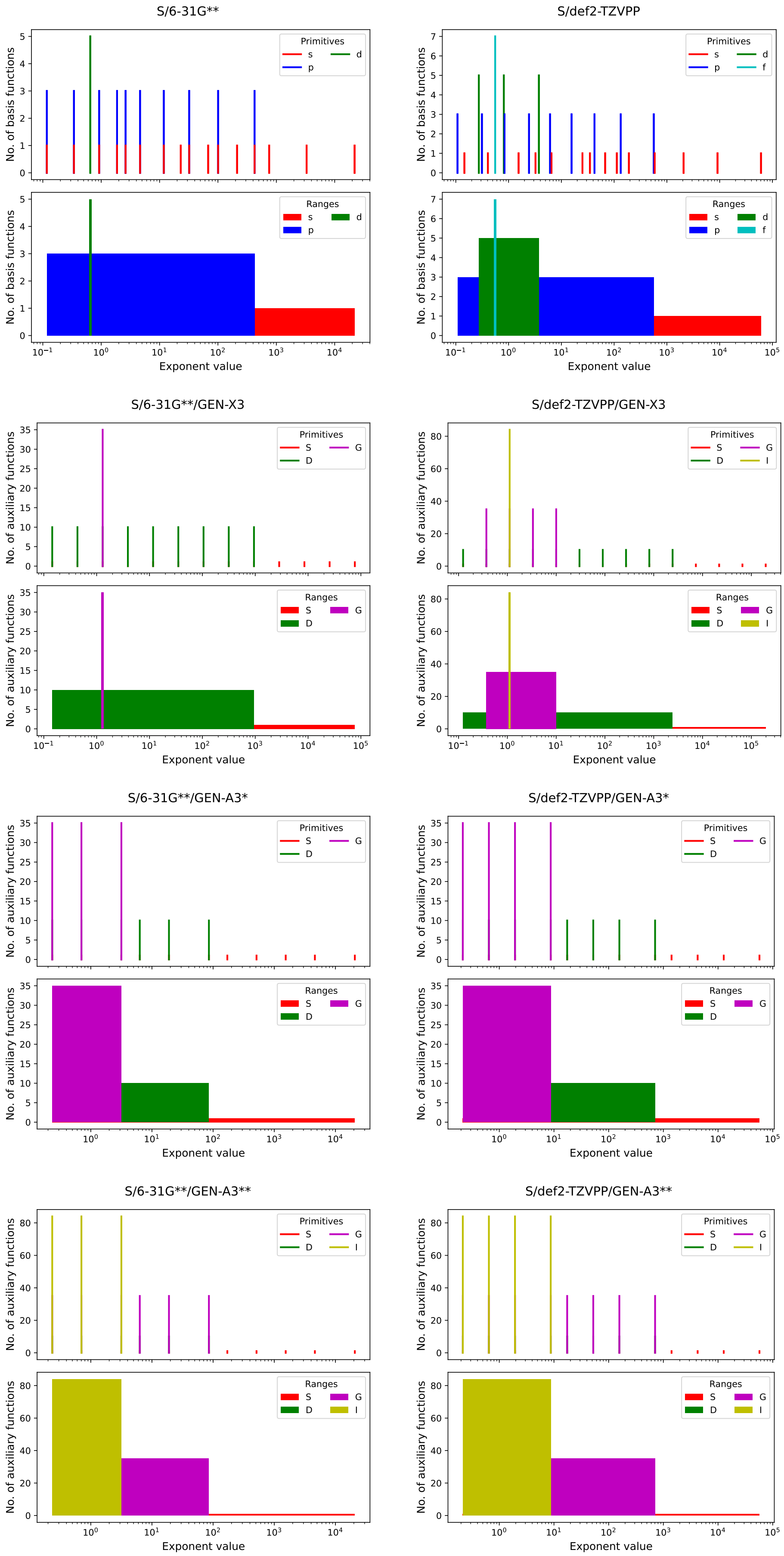

Figure S17: Plots for Cl

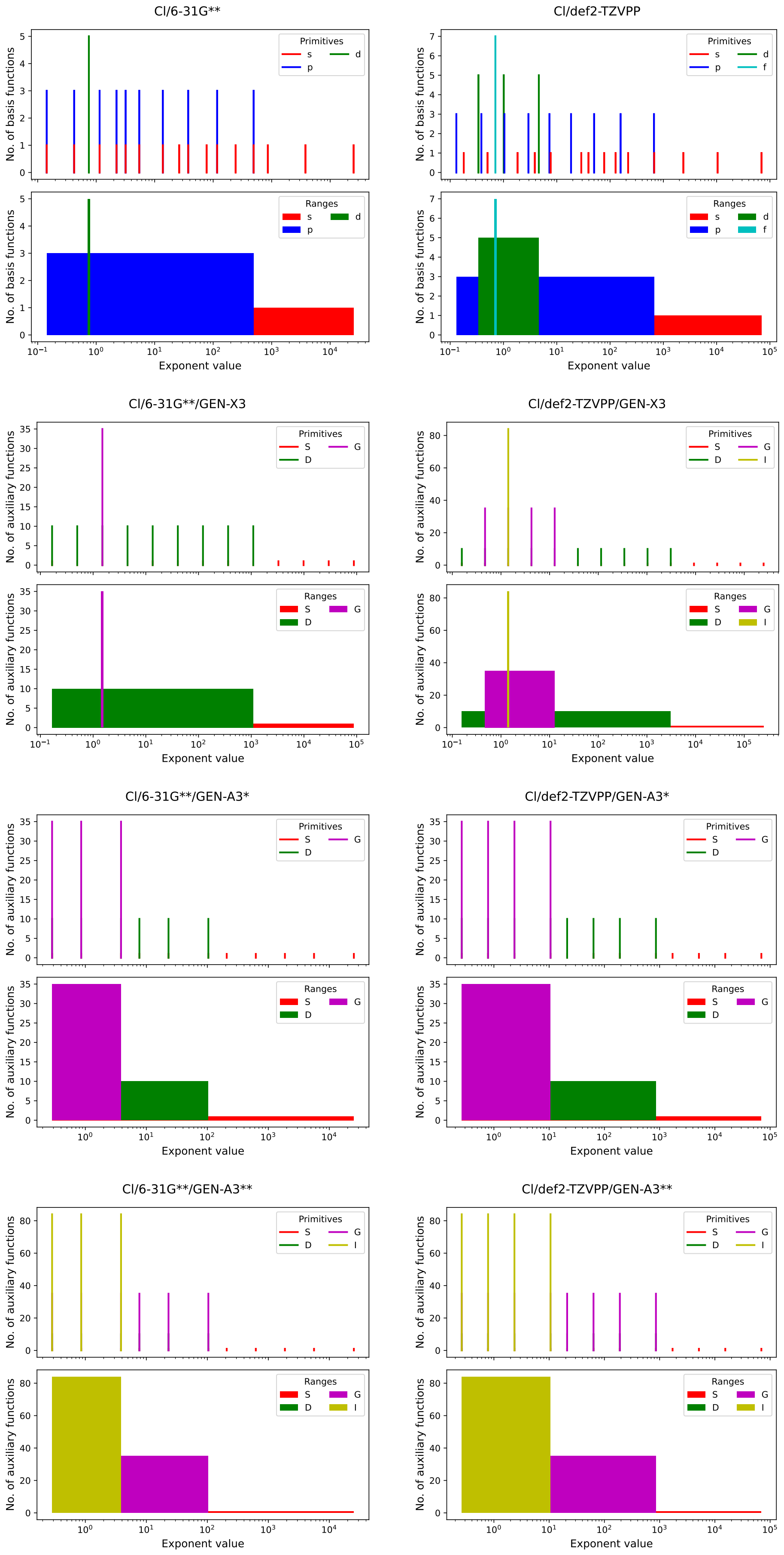

Figure S18: Plots for Ar

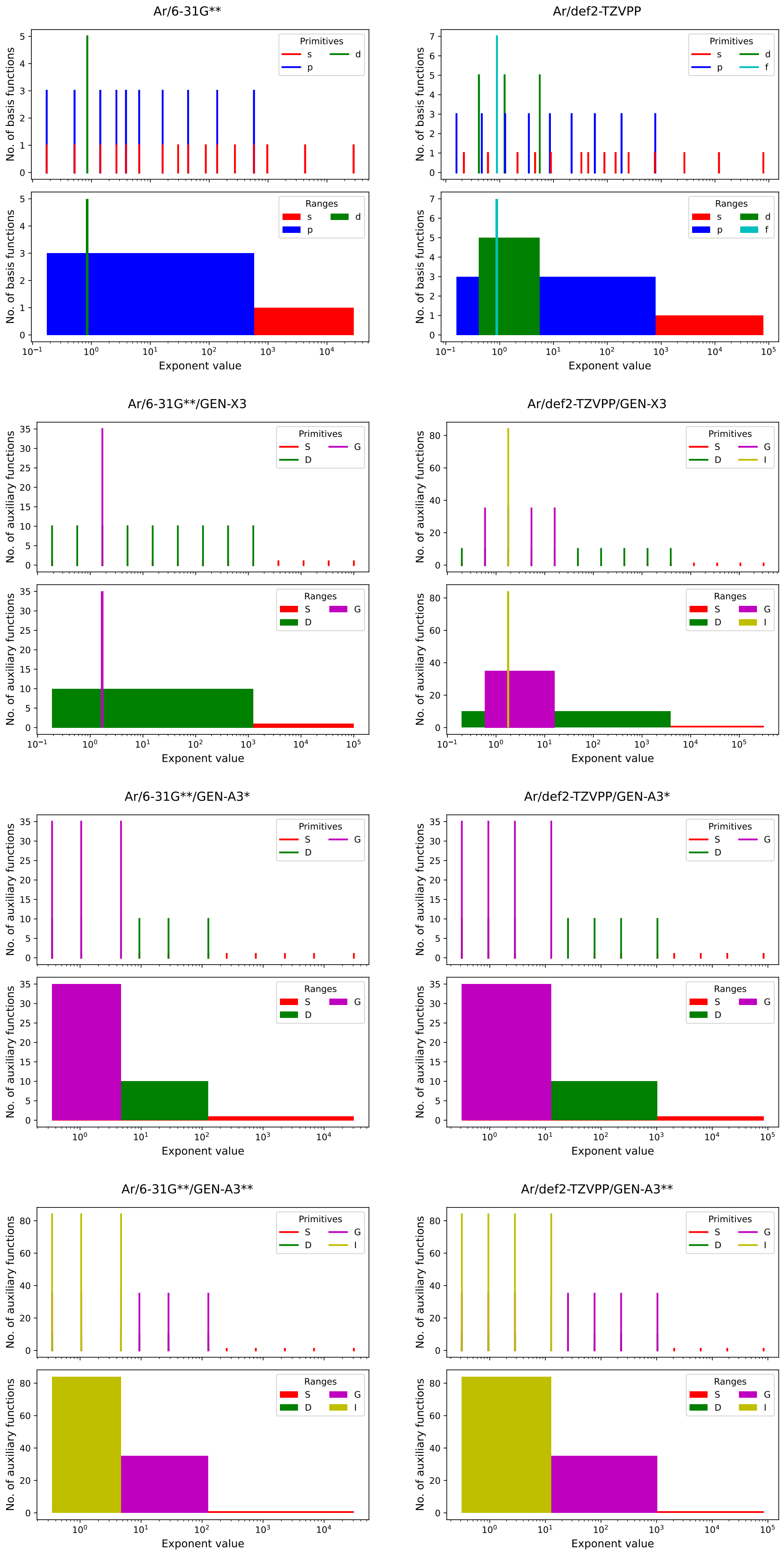

Figure S19: Plots for K

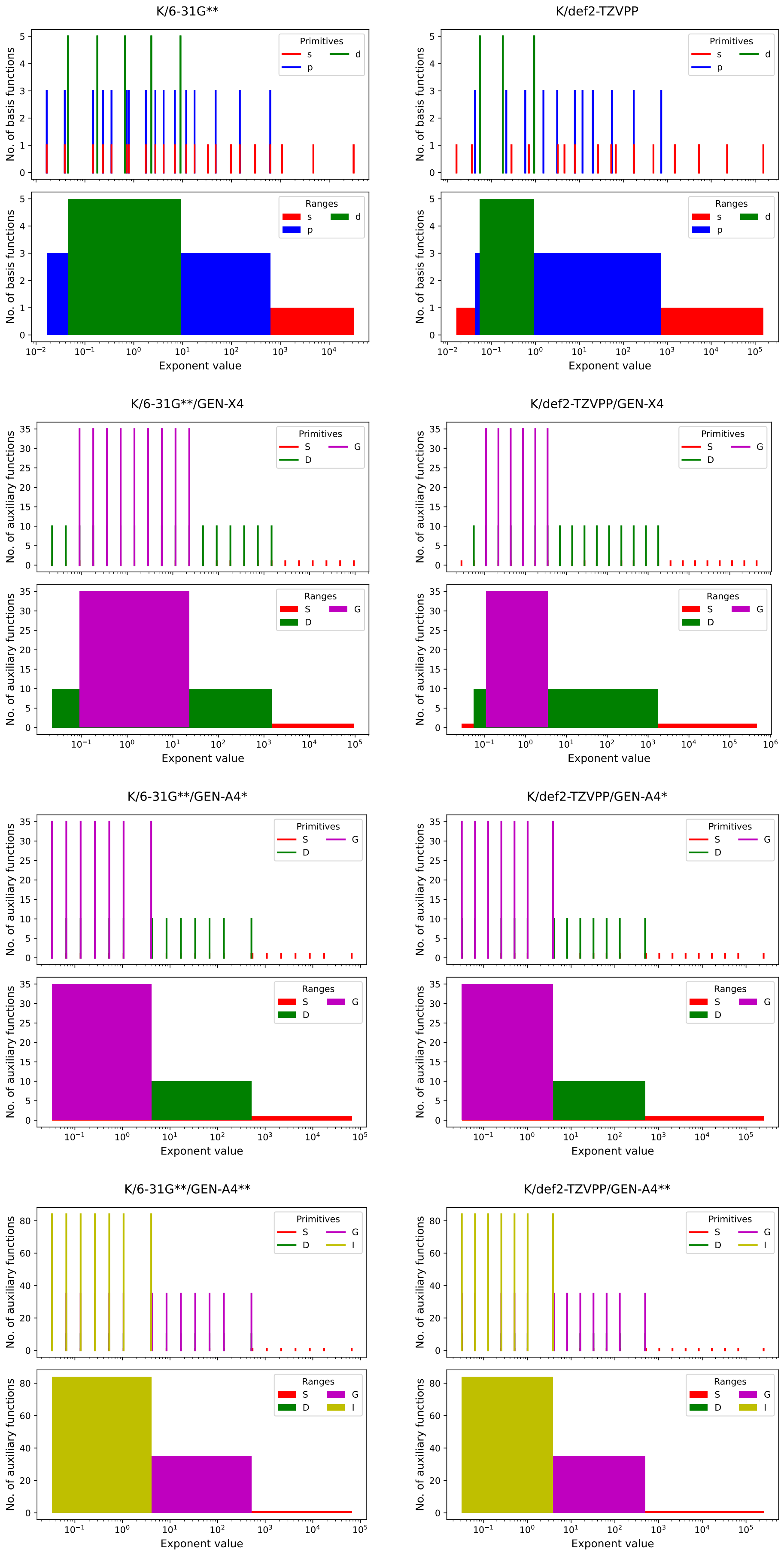

Figure S20: Plots for Ca

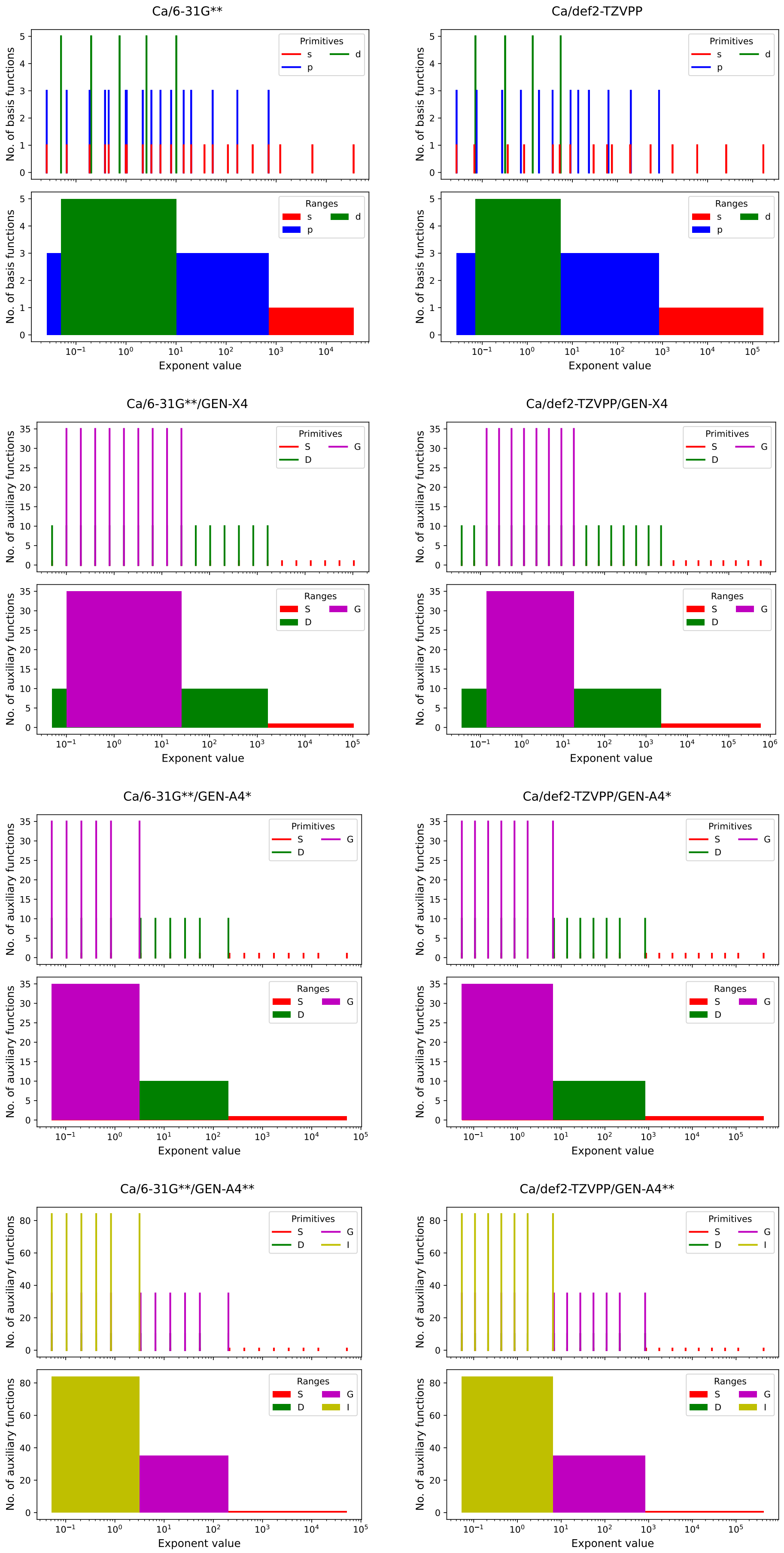

Figure S21: Plots for Sc

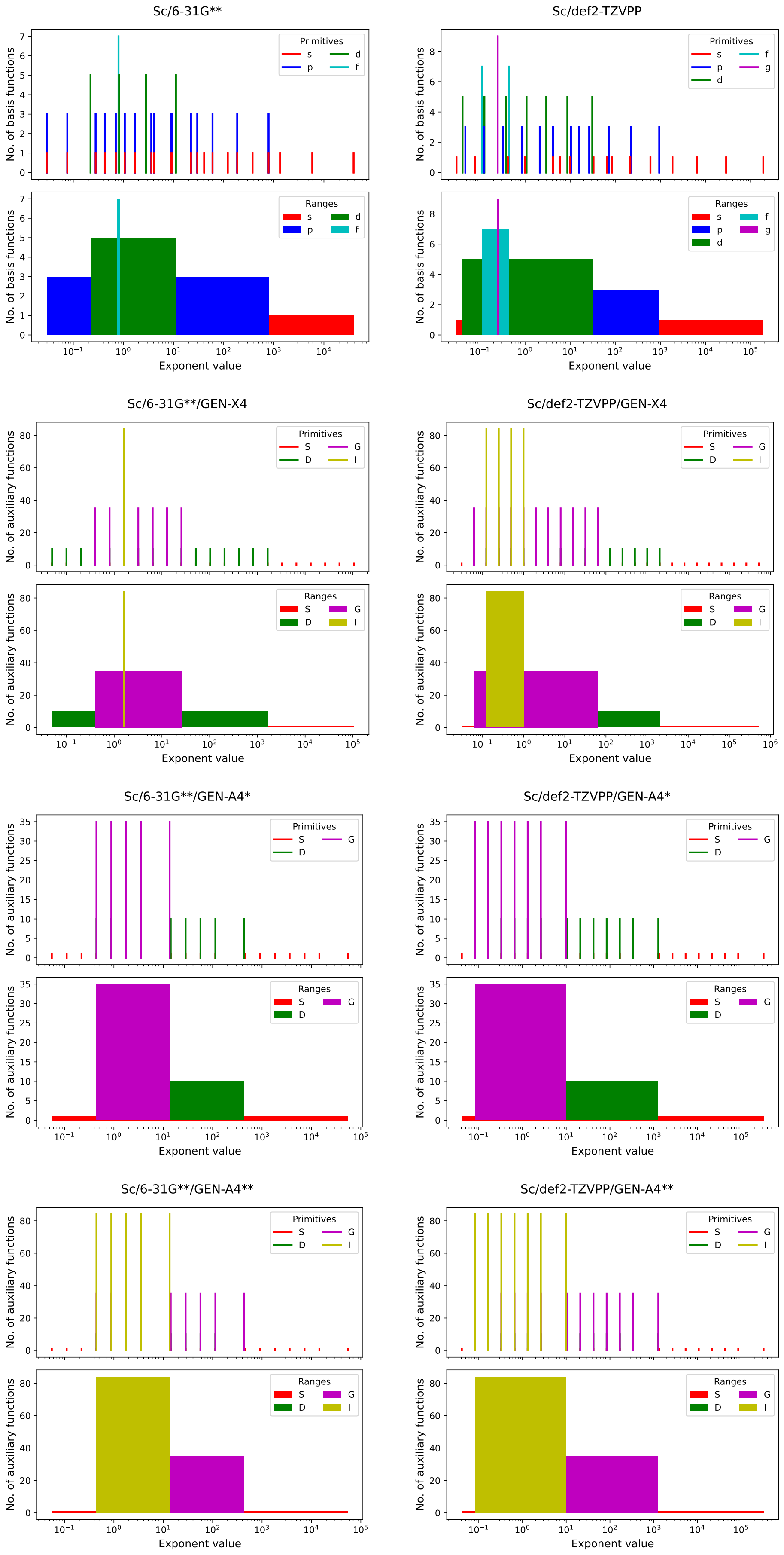

Figure S22: Plots for Ti

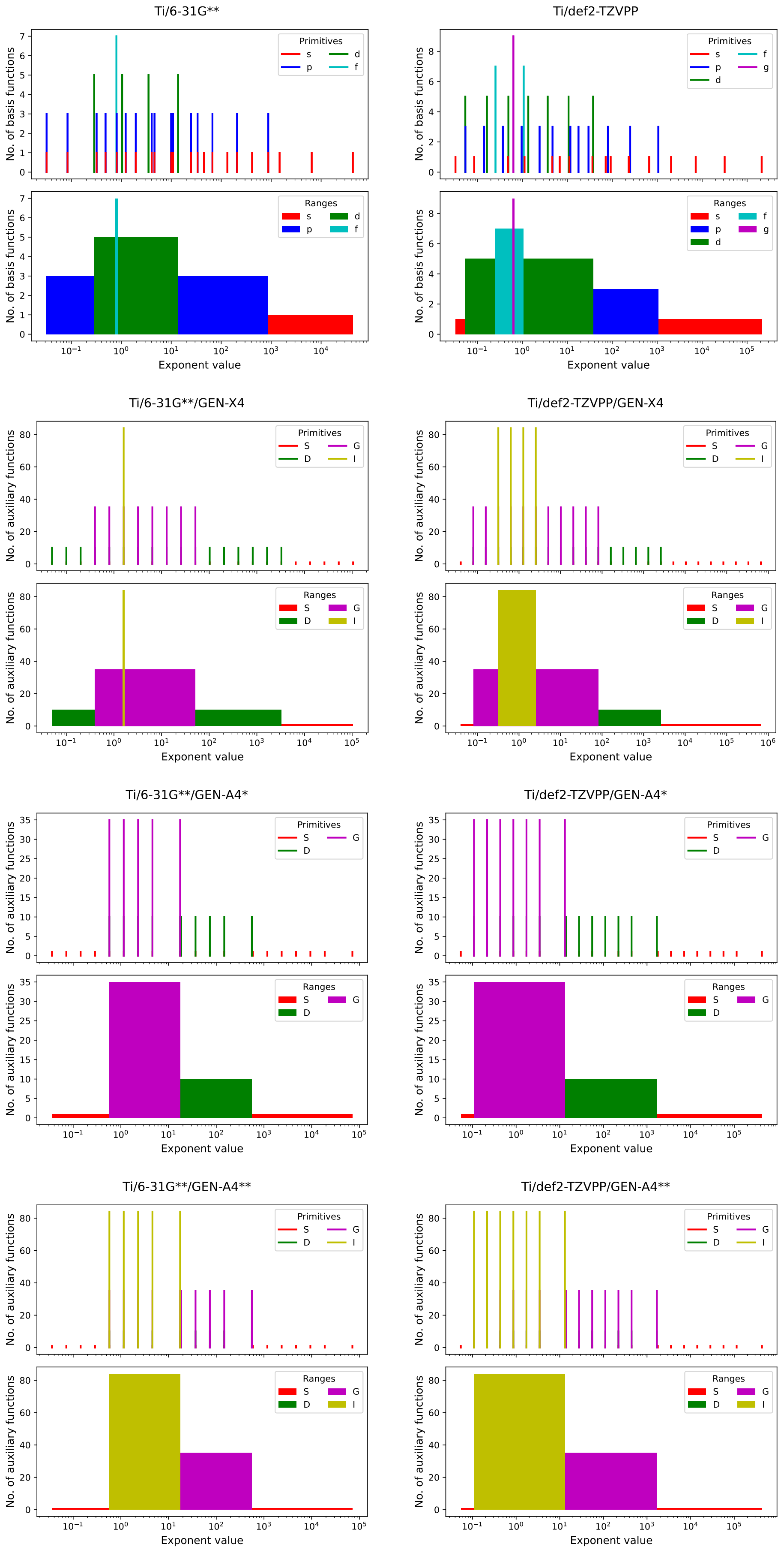

Figure S23: Plots for V

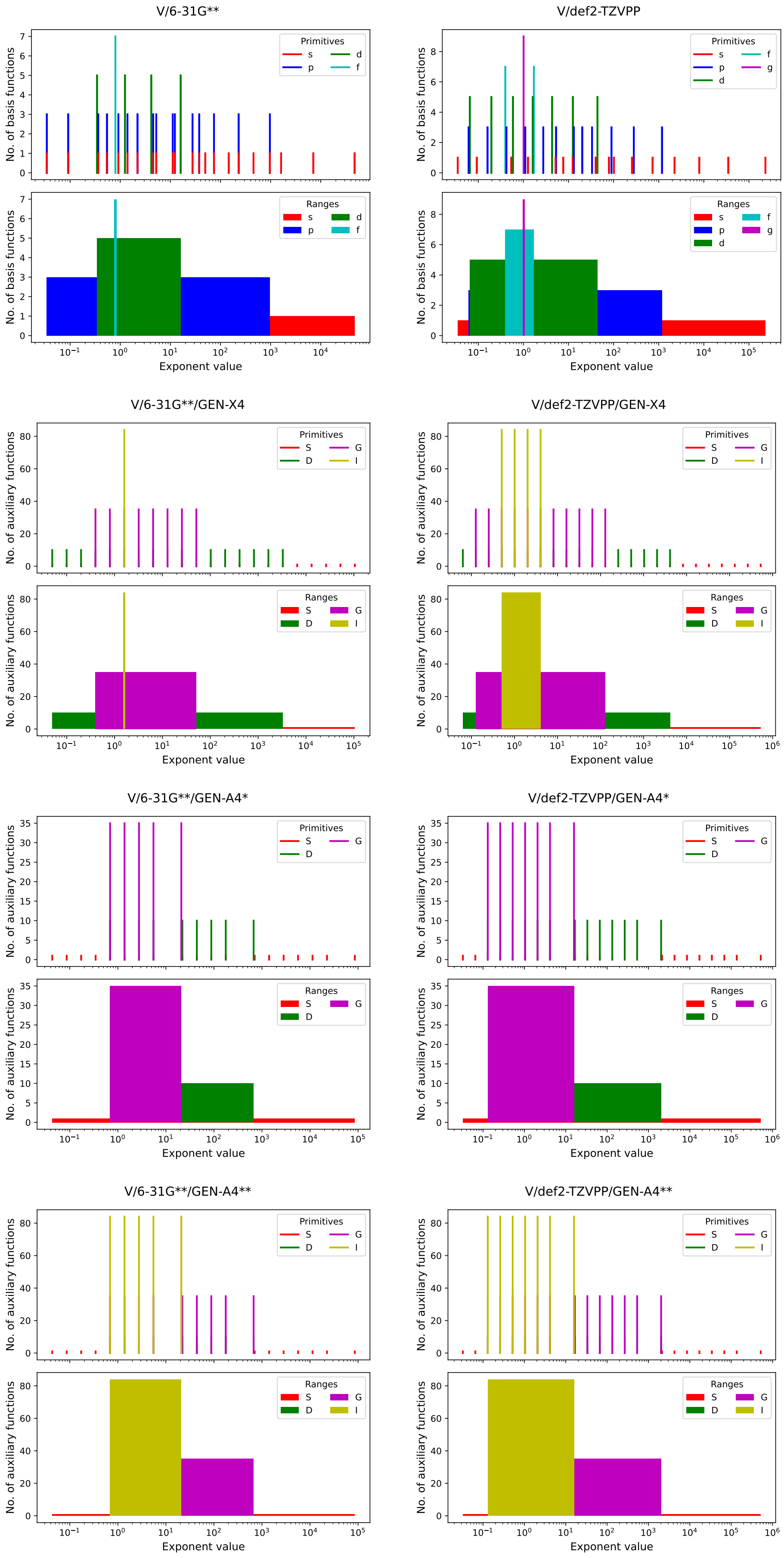

Figure S24: Plots for Cr

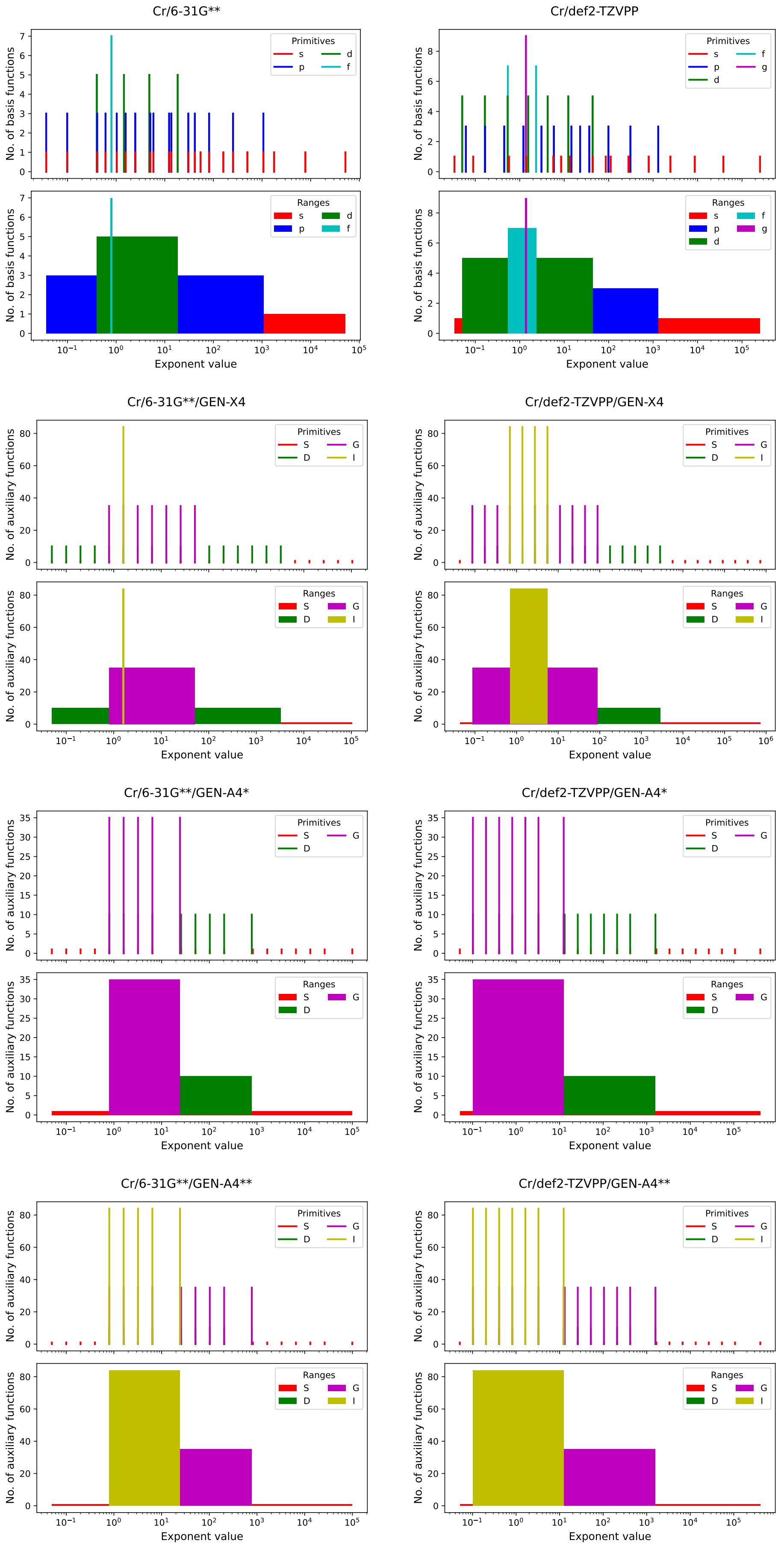

Figure S25: Plots for Mn

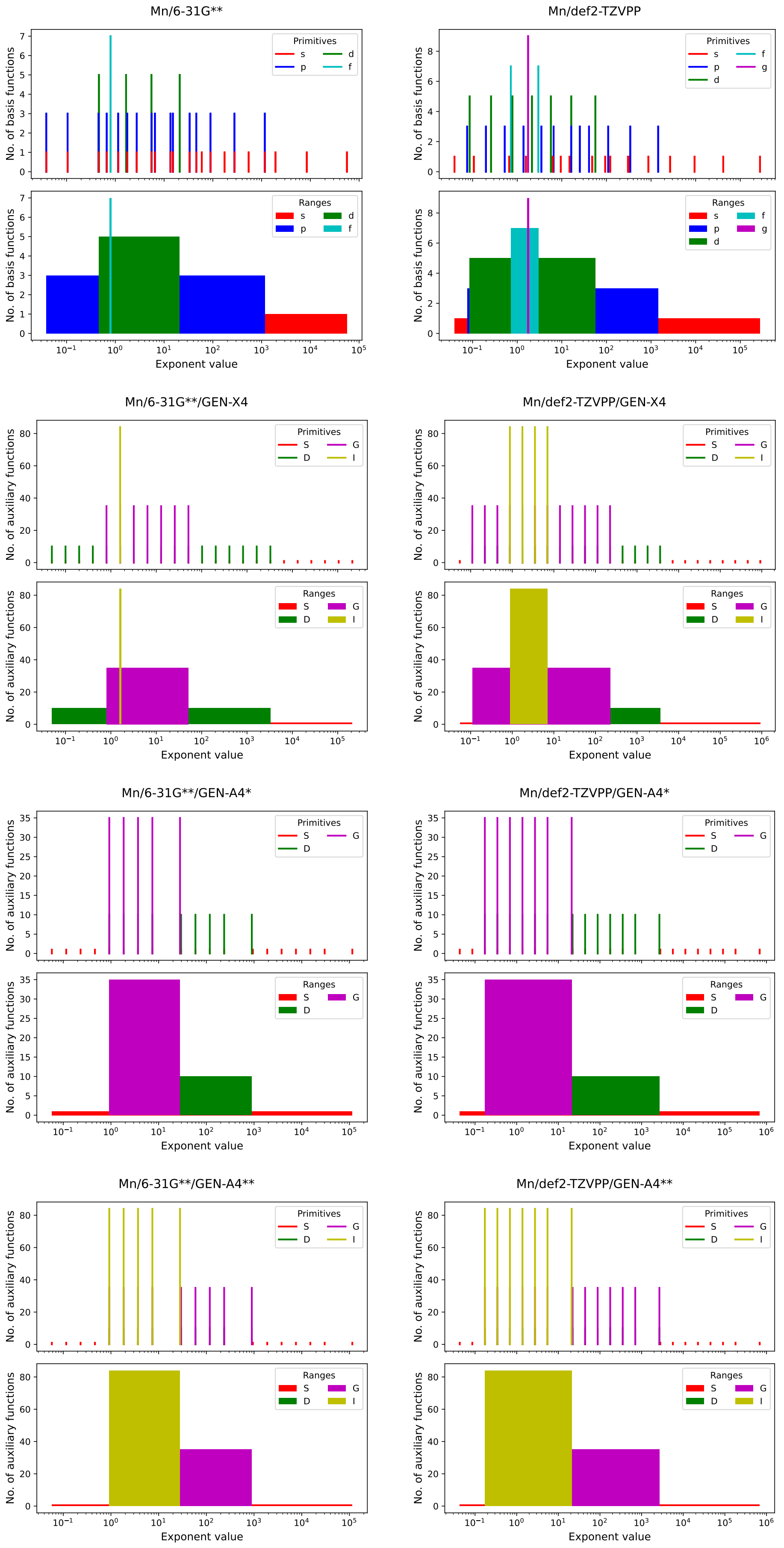

Figure S26: Plots for Fe

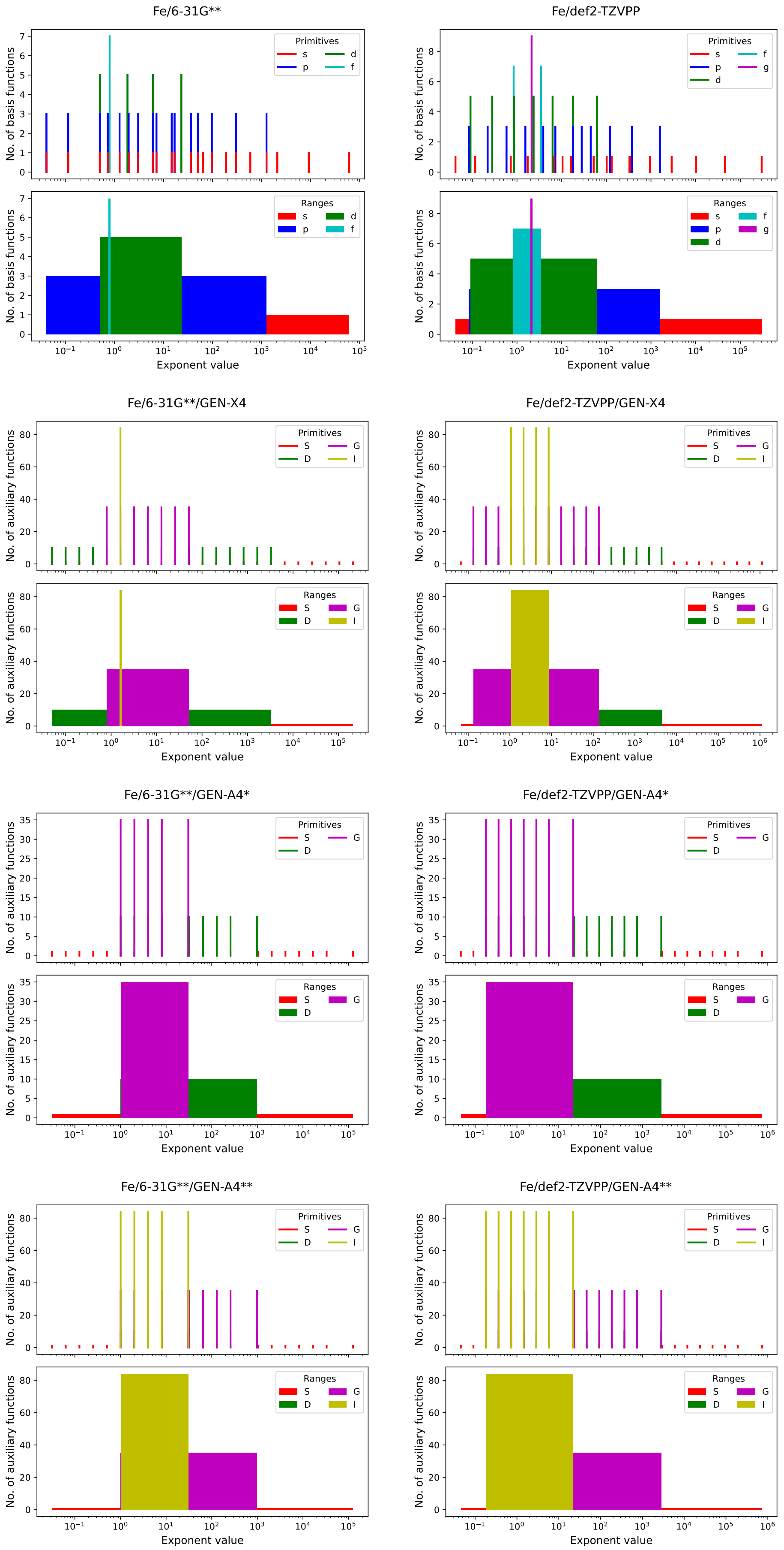

Figure S27: Plots for Co

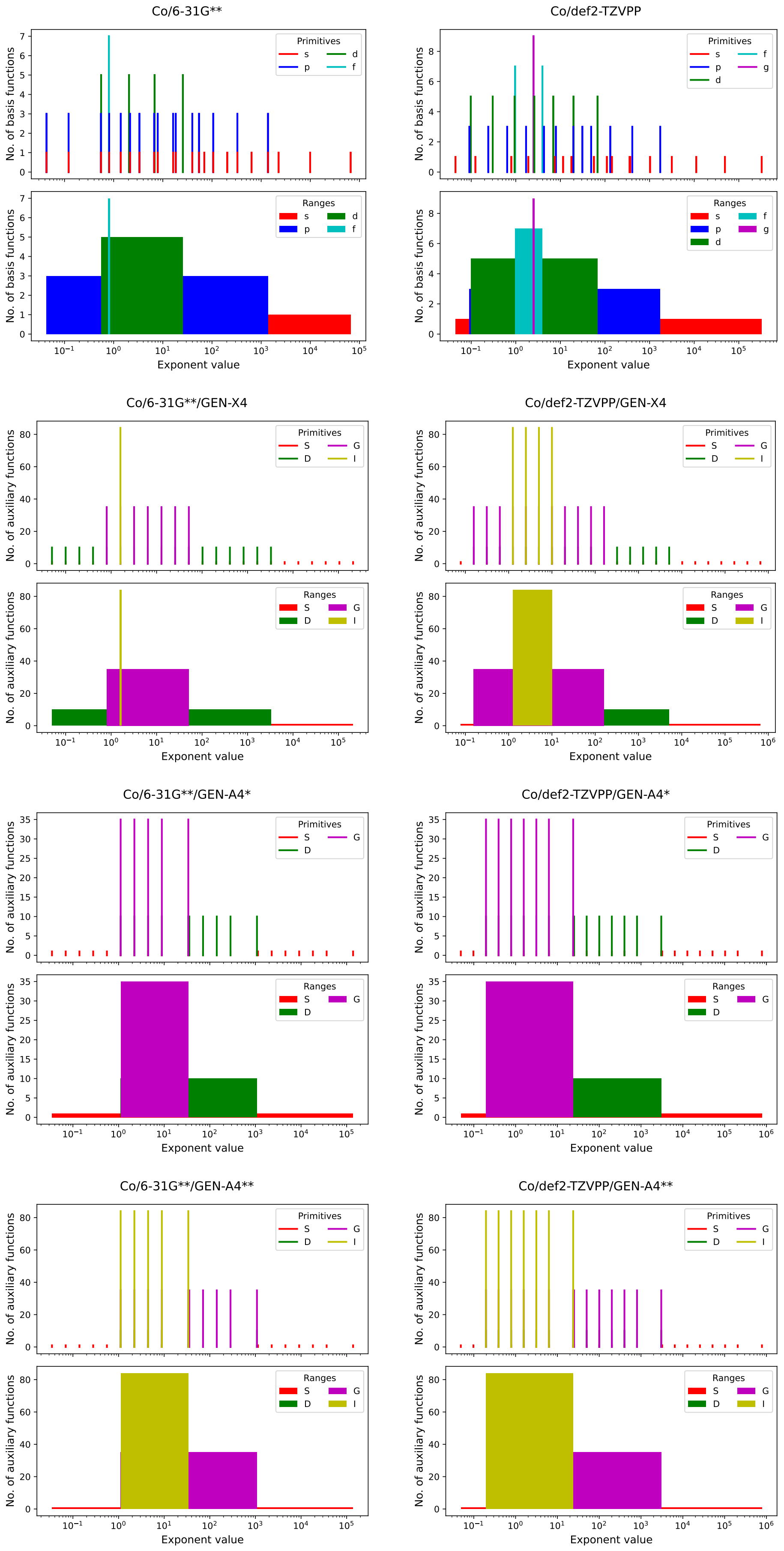

Figure S28: Plots for Ni

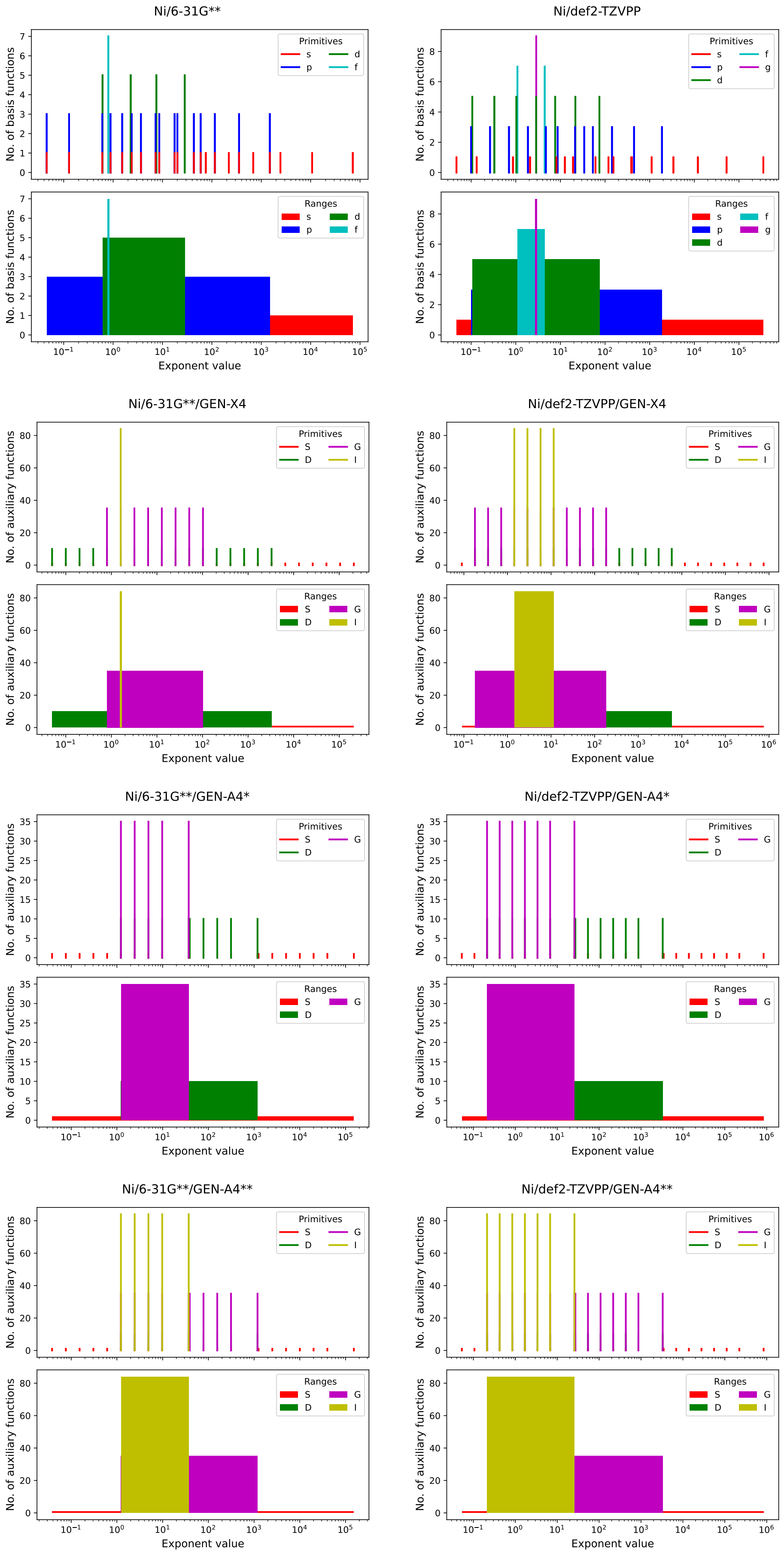

Figure S29: Plots for Cu

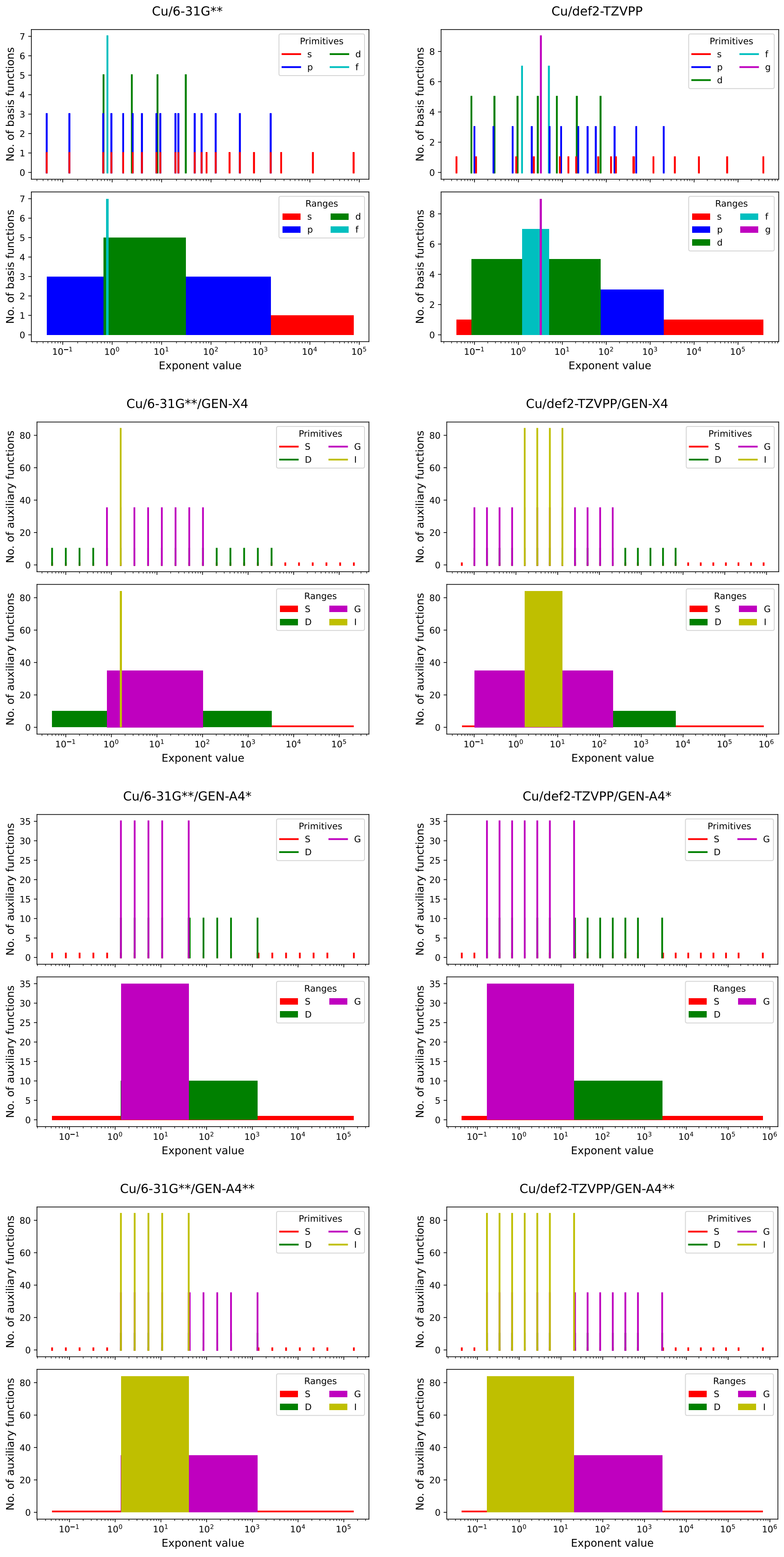

Figure S30: Plots for Zn

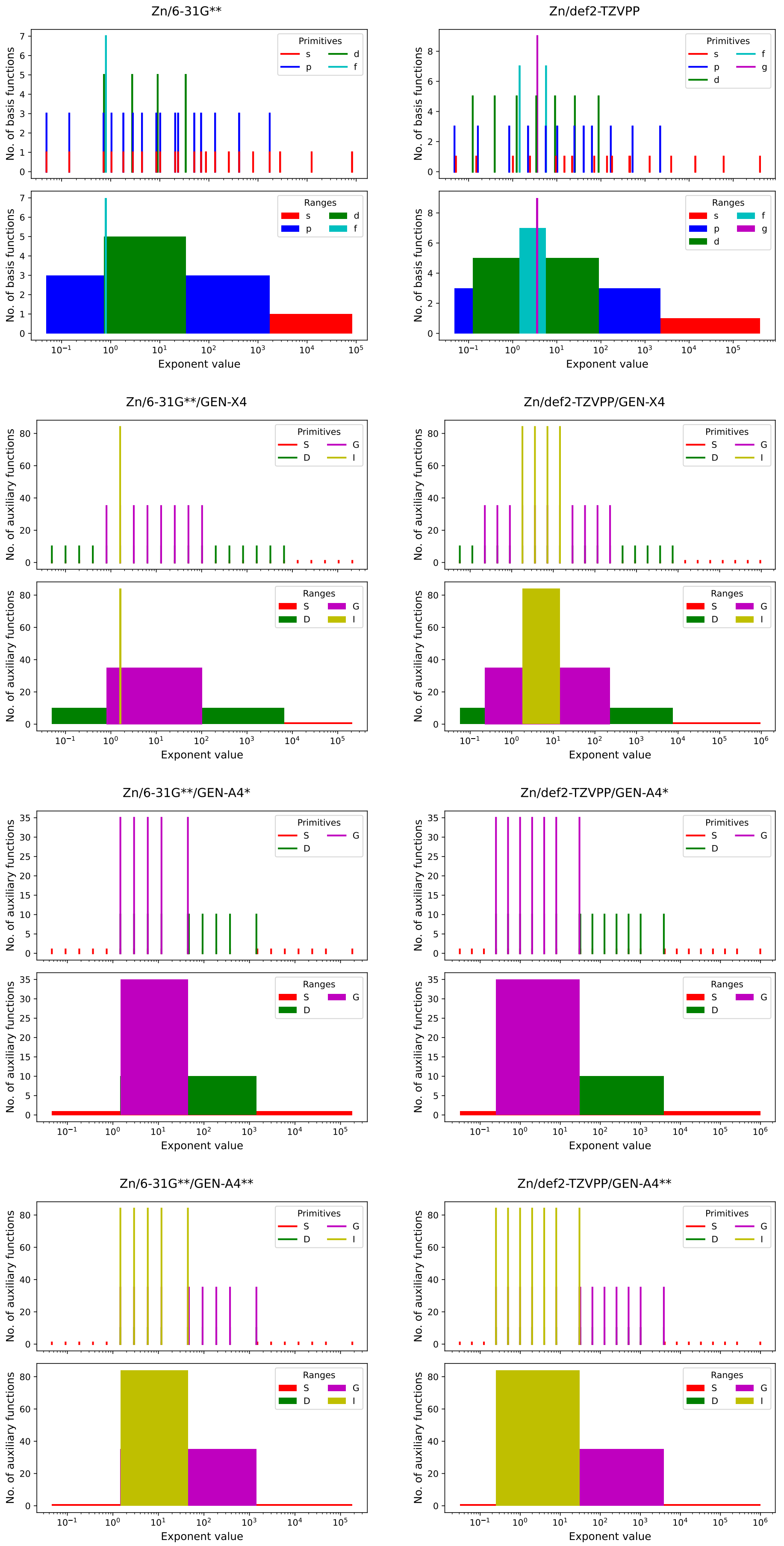

Figure S31: Plots for Ga

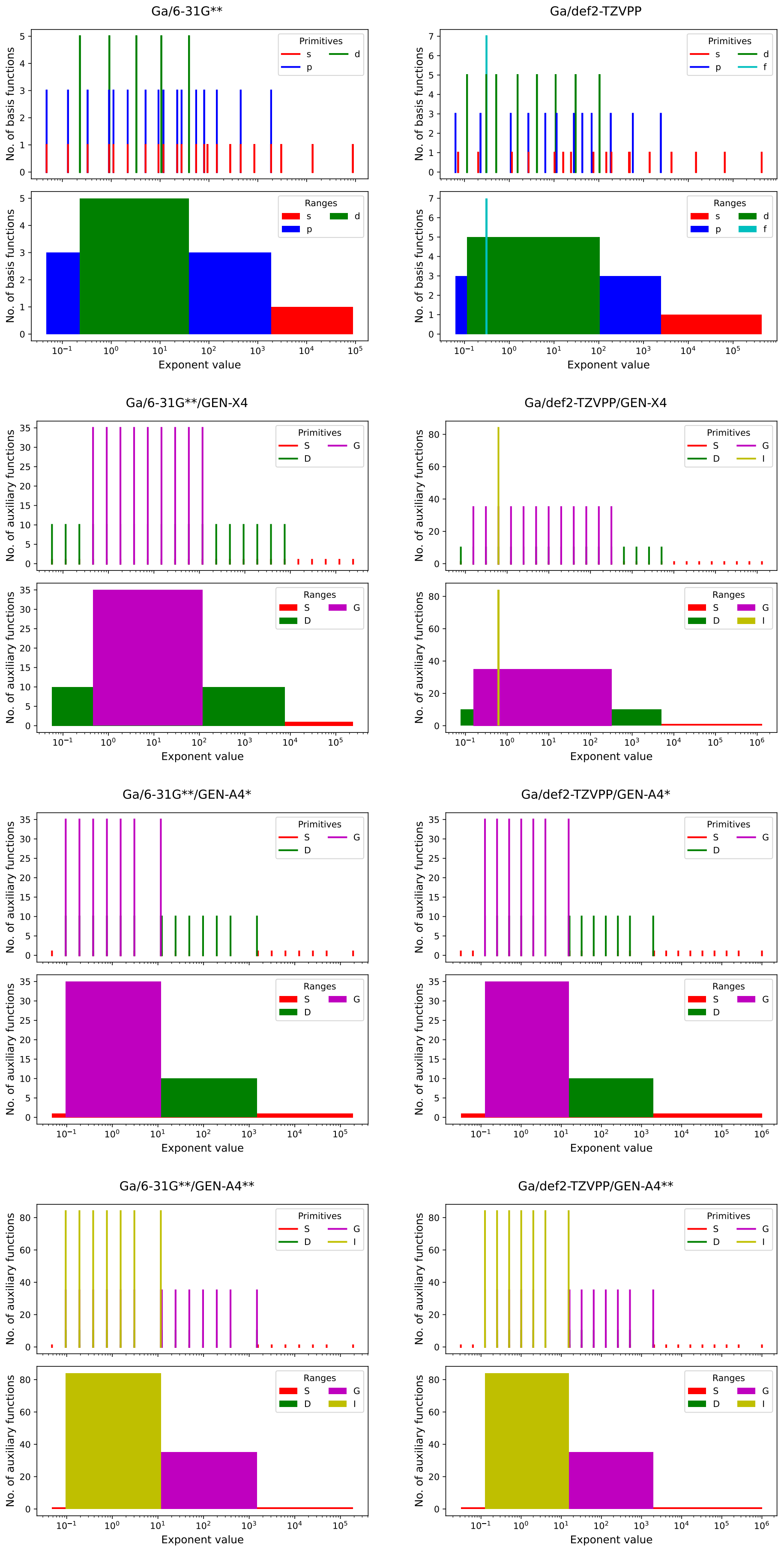

Figure S32: Plots for Ge

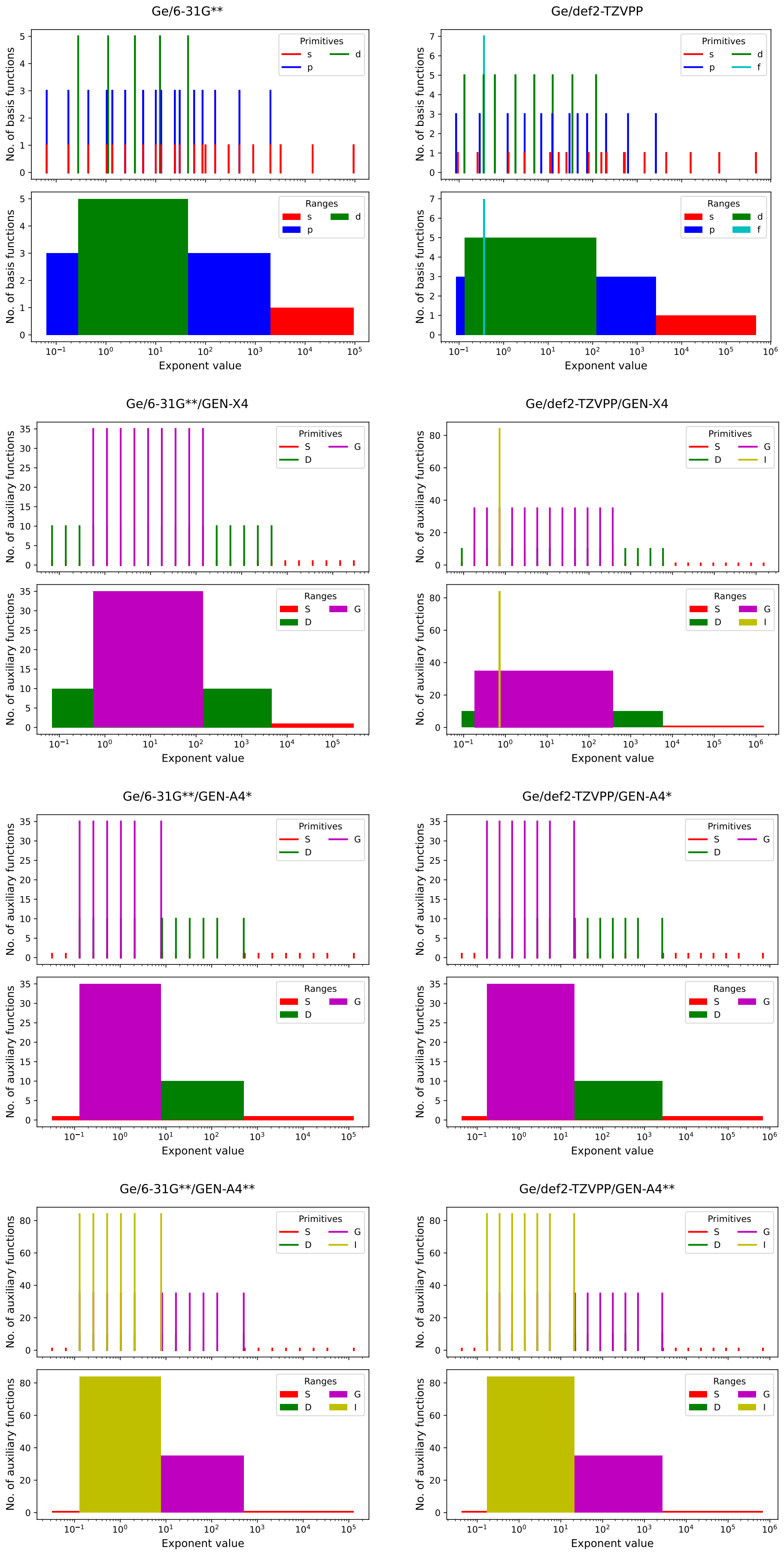

Figure S33: Plots for As

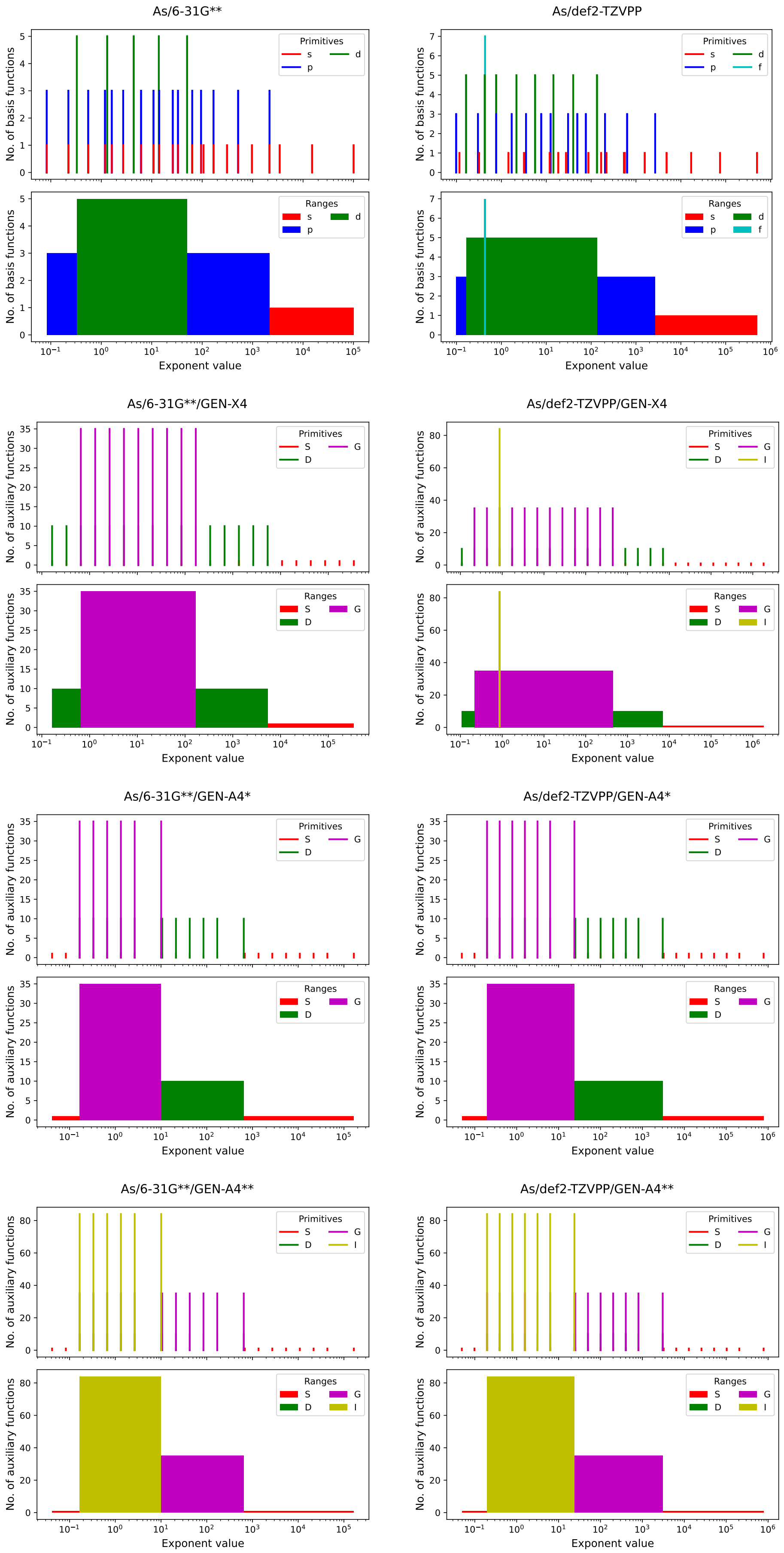

Figure S34: Plots for Se

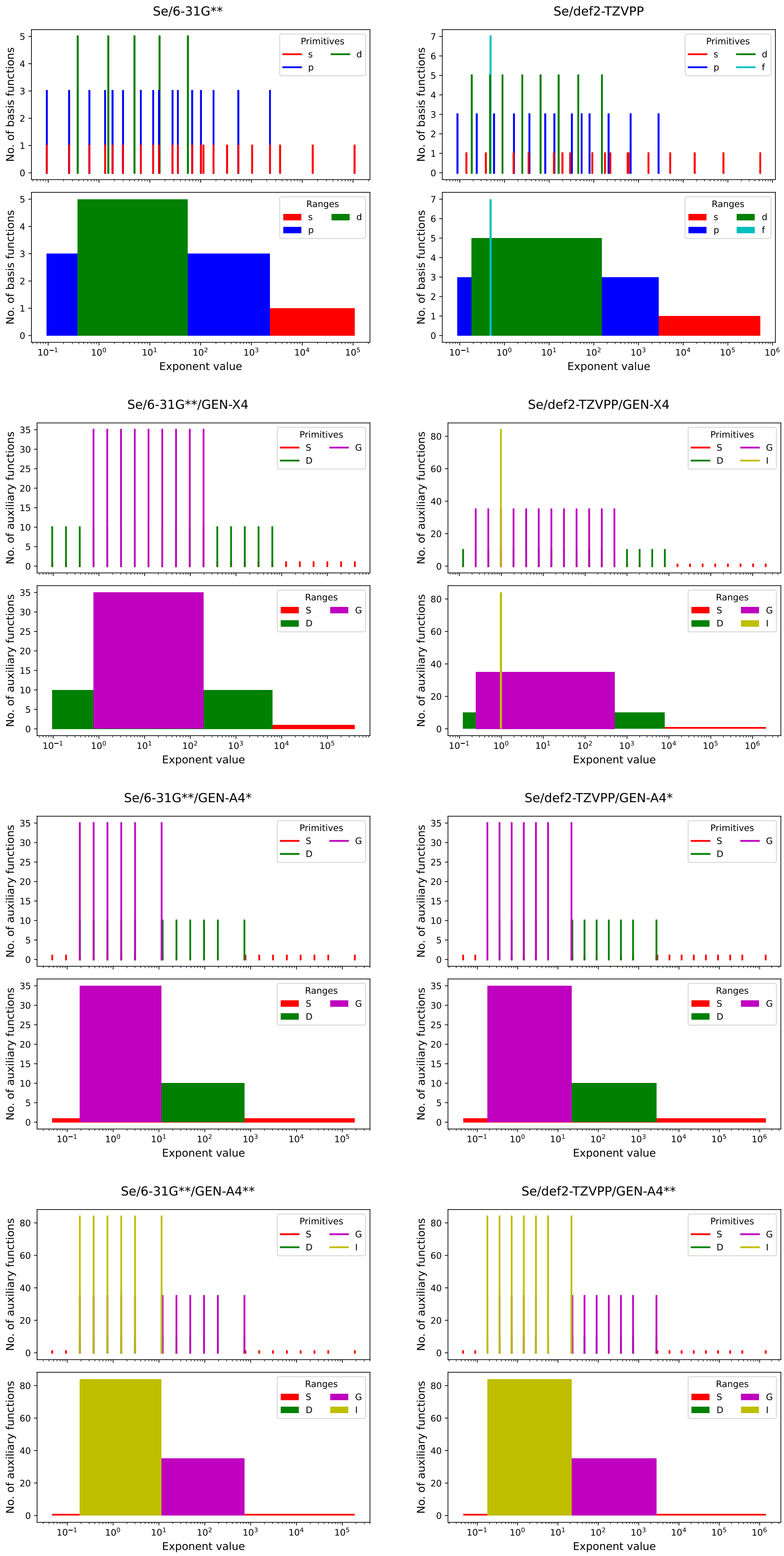

Figure S35: Plots for Br

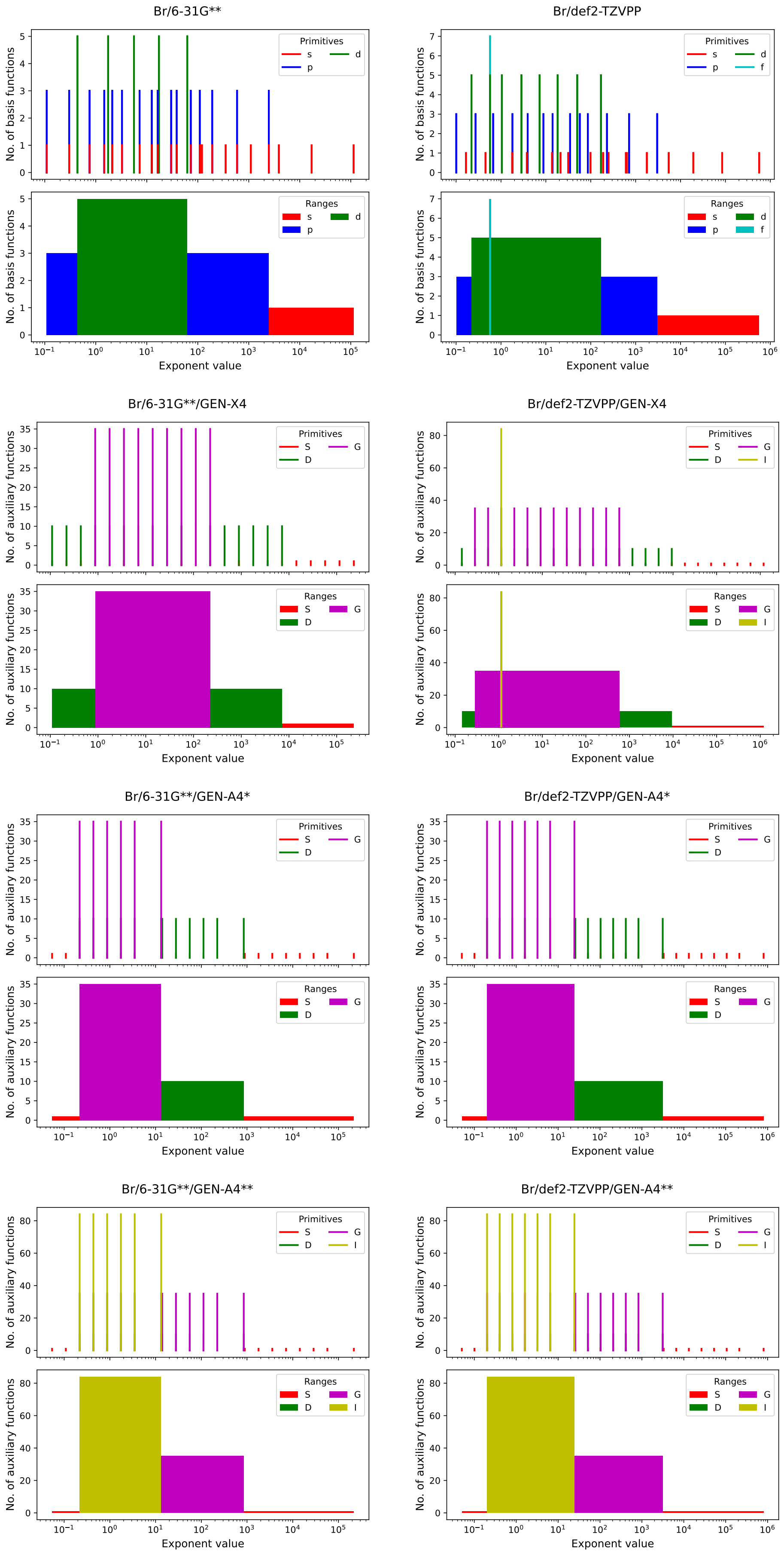

Figure S36: Plots for Kr

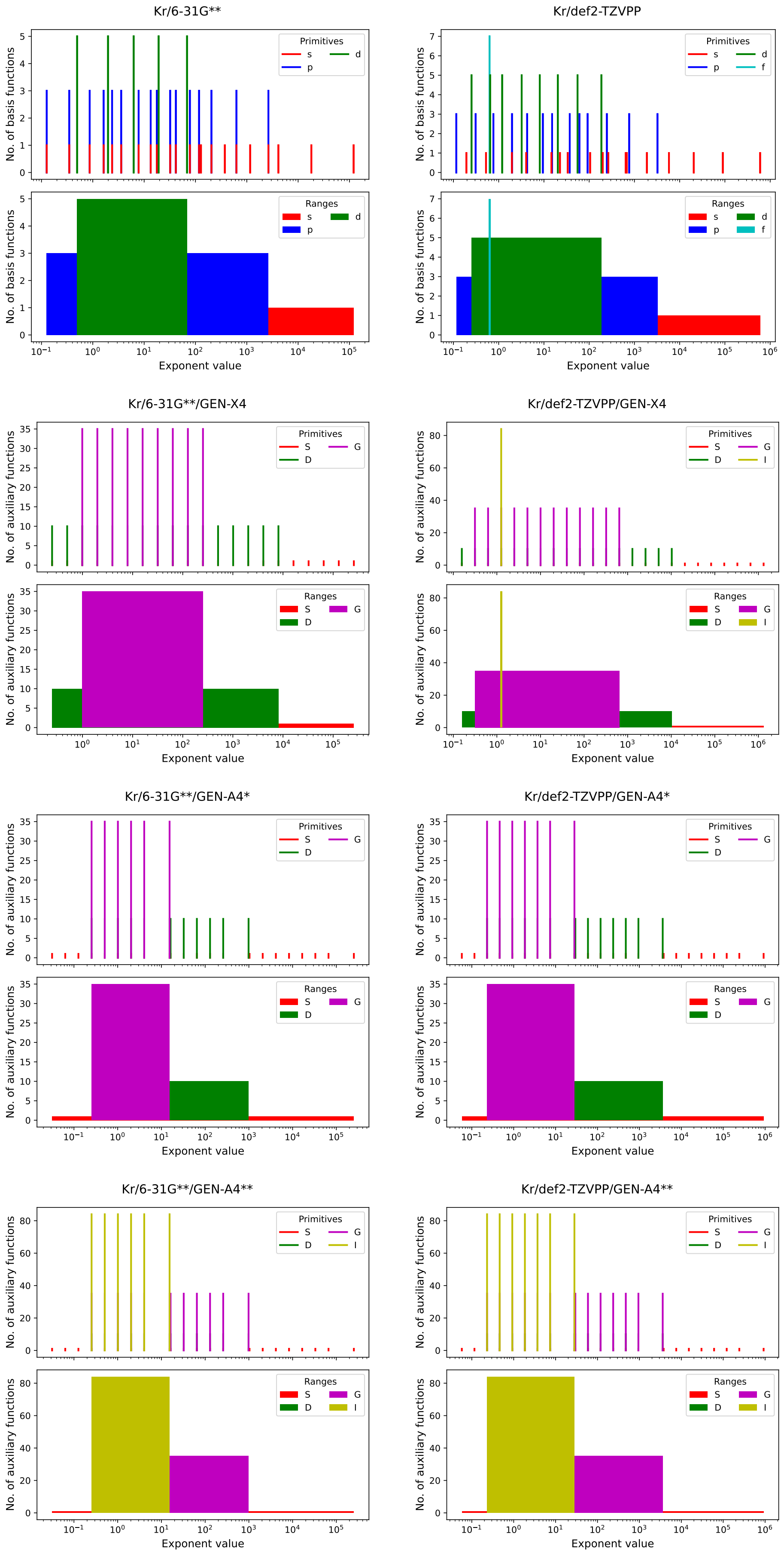

Table S1: Largest five signed SCF energy variational density fitting errors [kcal/mol] for def2-TZVPP orbital basis set and GEN-X4 auxiliary basis set in PBE, HF and PBE0 calculations of the T186 test set

PBE		HF		PBE0	
Molecule	MSE	Molecule	MSE	Molecule	MSE
Se_8	-0.20	Se_8	0.45	NiF_3	0.16
As_4S_4	-0.15	PF_5	0.43	Be_2F_4	0.12
As_4	-0.10	Li_4Cl_4	0.41	Se_8	0.12
$AsCl_6^-$	-0.09	$Ni(CO)_4$	0.40	TiF4	0.10
$HCBr_3$	-0.08	$Fe(CO)_5$	0.38	GeF_4	0.10
